# Evaluation of the trypanocidal and immunomodulatory effects of LDT409, a cardanol derivative from cashew nut shell liquid

**DOI:** 10.3389/fimmu.2026.1749250

**Published:** 2026-03-18

**Authors:** Juliana Magalhães Chaves Barbosa, Yasmin Pedra-Rezende, Tatiana Galvão Melo, Gabriel Melo de Oliveira, Andressa Souza de Oliveira, Natália Cipriano Monteiro, Luiz Antonio Soares Romeiro, Anissa Daliry, Kelly Salomão

**Affiliations:** 1 Laboratório de Biologia Celular, Instituto Oswaldo Cruz, Fundação Oswaldo Cruz, Rio de Janeiro, RJ, Brazil; 2 Laboratório de Fisiopatologia Clínica e Experimental, Instituto Oswaldo Cruz, Fundação Oswaldo Cruz, Rio de Janeiro, RJ, Brazil; 3 Laboratório de Ultraestrutura Celular, Instituto Oswaldo Cruz, Fundação Oswaldo Cruz, Rio de Janeiro, RJ, Brazil; 4 Laboratório de Desenvolvimento de Inovações Terapêuticas, Núcleo de Medicina Tropical, Universidade de Brasília, Brasília, DF, Brazil

**Keywords:** cardanol derivative, cashew nut shell liquid, Chagas disease, chemotherapeutic agent for Chagas disease, natural products, *Trypanosoma cruzi*

## Abstract

**Introduction:**

Chagas disease (CD), caused by *Trypanosoma cruzi*, is a neglected tropical illness with significant public health impact, particularly due to its cardiac manifestations. Current etiological treatments, benznidazole and nifurtimox, are limited by prolonged administration, adverse effects, and low cure rates, especially in chronic cases. Natural products, such as cashew nut shell liquid (CNSL), are a promising source of bioactive compounds with trypanocidal and immunomodulatory properties, and the added benefit of low production costs, a valuable advantage in addressing neglected tropical diseases. LDT409, a synthetic saturated cardanol derivative from CNSL, was designed as a fatty acid mimetic and partial agonist of peroxisome proliferator-activated receptors (PPARs), nuclear transcription factors that regulate lipid metabolism and attenuate inflammation by modulating immune responses. In CD, PPARs regulate host–parasite interactions by promoting anti-inflammatory immune polarization, which mitigates tissue-damaging inflammation but may also increase susceptibility to *T. cruzi* infection.

**Methods:**

In this study, we evaluated the trypanocidal activity and immunomodulatory effects of LDT409 using *in vitro* assays and an *in vivo* murine model of acute CD.

**Results:**

Our findings indicate that LDT409 treatment was associated with a reduction in peak parasitemia and with modulation of the host immune response, characterized by a shift toward a Th2/Th17 profile and attenuation of a Th1-driven pro-inflammatory response, concomitant with reduced cardiac damage. Notably, the underlying mechanisms of these effects, including the involvement of PPARs, remain to be clarified.

**Conclusion:**

Together, these observations suggest that LDT409 may represent a promising, cost-effective, and sustainable compound for further investigation in the context of Chagas disease.

## Introduction

Chagas disease (CD) is a neglected tropical illness caused by the protozoan *Trypanosoma cruzi*, representing a significant public health challenge due to its impact on mortality and physical disability (1). Historically endemic in Latin America, CD has affected an estimated 6 million individuals, with an annual incidence of 30,000 new cases. Furthermore, approximately 65 million people worldwide remain at risk of infection (2).

Clinically, CD is divided into two distinct phases: acute and chronic. During the acute phase, most individuals present mild, self-limiting symptoms that often go undetected in clinical practice (1). However, in some cases, the acute phase can progress to Chagas cardiomyopathy (CC), the manifestation most frequently associated with mortality. Patients with CC commonly exhibit electrocardiographic (ECG) abnormalities, along with lymphoid inflammatory infiltration, myocyte damage, and subsequent reparative fibrosis in cardiac tissue (1, 3).

The current etiological treatment for CD involves administering benznidazole (Bz) or nifurtimox, both developed in the 1960s (4). However, the use of these drugs requires prolonged periods of administration, which leads to adverse effects, such as peripheral neuropathy, skin reactions, and gastrointestinal disturbances. Additionally, the emergence of drug-resistant *T. cruzi* strains further undermines therapeutic efficacy, particularly in patients with chronic diseases (1).

Exploration of the pharmaceutical potential of natural products is a primary strategy in drug discovery, providing candidates that are often more cost-effective and less toxic than conventional therapies (5–7). The cashew tree (*Anacardium occidentale* L.) is notable among widely used medicinal plants. One of its main derivatives, cashew nut shell liquid (CNSL), has been used traditionally for centuries in South America, Africa, and Asia (8, 9). Although CNSL and its derivatives may have limited commercial value, their significant technological potential stems from their phenolic composition and diverse biological properties. These include anti-inflammatory, antimicrobial, antioxidant, antitumor, larvicidal, and insecticidal effects, underscoring their considerable therapeutic relevance (10–12). These substances are a natural source of phenolic compounds, such as anacardic acid, cardanol, cardol, and 2-methylcardol (13).

Based on these considerations, our investigation has focused on a novel compound, a saturated cardanol derivative from CNSL, designated LDT409. This compound can be efficiently synthesized from CNSL, a natural by-product of the cashew industry that is abundantly available in low- and middle-income countries (14) and has been characterized as a fatty acid mimetic due to its structural features, which include a 15-carbon aliphatic chain and a phenolic group. LDT409 was specifically designed to recognize the peroxisome proliferator- activated receptors (PPARs) (15).

PPARs are members of the steroid hormone receptor superfamily and function as ligand-dependent nuclear transcription factors that play a crucial role in regulating lipid metabolism and inflammation (16, 17). Therefore, PPARs have been consistently identified as relevant molecular targets for therapeutic intervention, especially in the management of inflammatory diseases such as obesity, diabetes, and Chagas cardiomyopathy (15–21).

In CD, the role of PPARs has been extensively studied as modulators of inflammation and host–parasite interactions (22). Activation of Peroxisome Proliferator-Activated Receptor Gamma (PPARγ) has demonstrated anti-inflammatory and cytoprotective effects in *in vitro* models of *T. cruzi* infection, including infected cardiomyocytes (22, 23). Additionally, both PPARγ and Peroxisome Proliferator-Activated Receptor Alpha (PPARα) contribute to macrophage reprogramming during infection, promoting a shift from a pro-inflammatory M1 phenotype to a regulatory M2 profile, which is characterized by reduced expression of inducible nitric oxide synthase (NOS_2_) and pro-inflammatory cytokines (24). In murine models of acute and chronic infection, treatment with 15-deoxy-Δ^12^,^14^-prostaglandin J_2_ (15dPGJ2), a natural PPARγ ligand, suppresses inflammatory mediators and matrix metalloproteinase activity, but is also associated with increased amastigote burden (16). Altered PPARγ expression in peripheral blood mononuclear cells from patients with chronic Chagas cardiomyopathy has also been linked to an unfavorable immune–metabolic profile (25).

Given these considerations and the profound impact of CD, particularly in developing countries, the urgent need for more effective compounds with fewer adverse effects, and the broad therapeutic potential of LDT409, it is strategically important to further investigate the pharmacological properties of this molecule as a potential candidate for CD treatment. Notably, the low production cost of LDT409 is an additional advantage, especially relevant in the context of neglected tropical diseases (26). Therefore, the main objective of this study was to assess the trypanocidal and immunomodulatory activities of LDT409 to advance its development as a novel therapeutic option.

## Methodology

### Physicochemical, ADMET, and potential molecular targets prediction

The physicochemical properties of LDT409 and its potential molecular targets in *Mus musculus* were predicted using the SwissADME program (27). ADMET parameters (absorption, distribution, metabolism, elimination, and toxicity) were obtained by entering the LDT409 molecular structure into the ADMETsar platform.

### Parasites

Bloodstream trypomastigotes (BT) of the Y strain of *T. cruzi* (DTU II) were obtained by cardiac puncture of infected Swiss Webster mice at the peak of parasitemia, followed by differential centrifugation (500 × g for 30 min at 4 °C). The parasites were resuspended in RPMI-1640 medium (Life Technologies™, Carlsbad, USA) supplemented with 10% mycoplasma-free, inactivated fetal bovine serum (FBS) (Cultilab™, Campinas, Brazil), 1 mM L-glutamine (Sigma-Aldrich™, St. Louis, USA), and 1% penicillin/streptomycin solution (Life Technologies™). The Y strain was previously classified as partially resistant to Bz, exhibits high virulence, and may induce cardiac disease and mega syndromes (28, 29).

### Drug

LDT409 was synthesized as previously described by Sahin et al. (2022) (15). For *in vitro* assays, a 100 mM stock solution of LDT409 was prepared in dimethyl sulfoxide (DMSO) (Sigma™), and aliquots were stored at -20 °C. The final concentration of the solvent in the assays did not exceed 0.6%, which does not cause any toxicity (30). For *in vivo* experiments, LDT409 was diluted in a vehicle solution containing 10% DMSO (v/v), 20% PEG400 (v/v) (Êxodo Científica, São Paulo, Brazil), 3% Tween 80 (v/v) (Sigma™), and sterile distilled water. All final concentrations used for preparing these vehicles do not cause detectable toxic effects in mice, as assessed by behavior and clinical parameters.

### Activity against bloodstream forms of *T. cruzi*

For the *in vitro* assays, BT forms (5×10^6^ cells/mL) were incubated at 37 °C in a 5% CO_2_ atmosphere in the absence or presence of LDT409 at serial concentrations up to 1000 µM. After 24 h of incubation, parasite counts were performed in a Neubauer chamber under light microscopy (Zeiss™, Oberkochen, Germany). The activity of the compounds was expressed as the EC_50_/24 h, corresponding to the concentration that resulted in 50% lysis of the parasites during the 24 h period.

### Activity against intracellular forms of *T. cruzi*

Evaluation of the activity of LDT409 against intracellular forms was performed using primary cultures of 18-day-old mouse embryo heart cells (HMCs) (31). The HMCs were obtained as described by Barbosa et al. (2022) (32), and cultured in Dulbecco’s Modified Eagle Medium (DMEM) (Life Technologies™) supplemented with 10% fetal bovine serum (FBS) (Cultilab™), 2.5 mM CaCl_2_ (Sigma™), 1 mM L-glutamine (Sigma™), 2% chicken embryo extract, and 1% penicillin/streptomycin solution (Life Technologies™). Cells were plated in 24-well plates at a density of 1.5 x 10^5^ cells/well on glass coverslips coated with 0.01% gelatin (Sigma™) and maintained at 37 °C in a 5% CO_2_ atmosphere. HMCs were infected with BT at a multiplicity of infection (MOI) of 10:1 (parasites/host cells) in 500 µL of supplemented DMEM-FBS. After 24 h, the cultures were washed with phosphate buffer saline (PBS) 1_X_ (Sigma™) to remove non-adherent parasites and incubated for 24 or 48 h at 37 °C in a 5% CO_2_ atmosphere in the absence or presence of the compound at serially diluted non-toxic concentrations (up to 100 µM), maintaining a final volume of 1 mL in each well. Following treatment, the cultures were rinsed with saline, fixed, and stained with Diff-Quick Staining (Laborclin™, Paraná, Brazil). The percentage of infection was determined by randomly counting at least 200 cells per coverslip under light microscopy. The results were expressed by the infection index (II), calculated by multiplying the percentage of infected cells by the number of parasites per infected cell (32) (**Supplementary Figure 1**). The EC_50_ values were calculated for each time point, corresponding to the concentration required to achieve 50% inhibition of the II (EC_50_ II).

### Mammalian cells cytotoxicity evaluation

Non-infected HMCs were incubated at 37 °C for 24 and 48 h with increasing concentrations of LDT409 (32 to 500 µM; 1:2 serial dilutions). After treatment, PrestoBlue™ (Invitrogen™, Carlsbad, USA) was added at a 1:10 ratio (PrestoBlue™: medium), the microplates were incubated for 2 h, and fluorescence was measured at 560 and 590 nm, as recommended by the manufacturer, using a Spectra Max™ M3 spectrofluorometer (Molecular Devices™, Sunnyvale, USA). Results were expressed as the difference in the percentage of reduction in viability between treated and untreated cells. The LC_50_ value was defined as the concentration that caused 50% loss of viability in mammalian cells. The selectivity index (SI) was calculated as the ratio of LC_50_ and EC_50_ at 24 h and 48 h in experiments with intracellular amastigotes (32).

### Scanning electron microscopy

BT forms of *T. cruzi* were treated for 24 h with LDT409 at concentrations corresponding to the IC_50_/24 h. The parasites were then fixed in 2.5% glutaraldehyde in 0.1 M sodium cacodylate buffer (pH 7.2) for 40 min at 25 °C, followed by post-fixation in 1% osmium tetroxide (OsO_4_), 0.8% potassium ferricyanide, and 2.5 mM calcium chloride (CaCl_2_) in the same buffer for 20 min at 25 °C. Subsequently, the cells were dehydrated in an ascending ethanol series, dried by the critical point method with CO_2_, mounted on aluminum stubs, coated with an approximately 20 nm thick gold layer in a Sputter Coater 108 (Cressington Scientific Instruments, Watford, UK), and examined on a Jeol JSM6390LV scanning electron microscope™ (Jeol, Tokyo, Japan) located in the Rudolf Barth Electron Microscopy Platform (Instituto Oswaldo Cruz, Fiocruz, Rio de Janeiro, RJ, Brazil).

### Transmission electron microscopy

BT of *T. cruzi* were treated for 24 h, with LDT409 concentrations corresponding to the IC_50_. The cells were fixed and post-fixed, as described for SEM analysis. They were dehydrated in an ascending acetone series and embedded in Polybed 812 resin™. Ultrathin sections were stained with uranyl acetate and lead citrate and examined with a Hitachi HT7800 transmission electron microscope (Hitachi High-Tech Corporation, Tokyo, Japan) at the Rudolf Barth Electron Microscopy Platform (Instituto Oswaldo Cruz, Fiocruz, Rio de Janeiro, RJ, Brazil).

### *In vivo* assays

Five-week-old male Swiss Webster outbred mice were obtained from the animal facility of the Instituto de Ciência e Tecnologia em Biomodelos (ICTB, Rio de Janeiro, Brazil). The animals were housed five per cage in a conventional room at 20 to 24 °C under a 12 h/12 h light/dark cycle, with sterilized water and chow provided *ad libitum.* All procedures were conducted in accordance with the guidelines established by the FIOCRUZ Committee of Ethics for the Use of Animals (license L-012/2022-A2). An inhalation anesthesia system (Bonther Equipamentos, São Paulo, Brazil) was used for animal euthanasia. The animals were placed in an acrylic induction chamber, and anesthesia was induced with 3% isoflurane for 5 minutes. Subsequently, the isoflurane concentration was increased to 10% (overdose level) and maintained until cessation of respiratory movements and cardiac activity was observed, confirming death.

### Mouse infection and treatment schemes

Male mice were inoculated intraperitoneally (i.p.) with 10^4^ *T. cruzi* BT of the Y strain (DTU II) and subsequently treated by gavage. Following previous studies, we administered a short course of treatment for 5 consecutive days (once or twice daily), starting on the 5^th^ day postinfection (dpi), which corresponds to the onset of parasitemia in this experimental acute model (33). LDT409 was given at a dose of 100 mg/kg/day, selected based on previous results as reported in Sarin et al. (2022) (15). In that study, oral administration of LDT409 in a murine model demonstrated rapid plasma elimination, with concentrations approaching negligible levels within 12–16 h after oral administration (15). Based on this pharmacokinetic profile, we selected two treatment regimens: once daily (24-h interval; LDT409_1x_) and twice daily (12-h interval; LDT409_2x_). The aim was to maintain detectable plasma drug concentrations and to evaluate whether sustained levels could enhance the biological effect of the compound.

The two control groups were NI (non-infected mice, negative control) and Tc (infected mice, positive control), both receiving only the vehicle. Only animals confirmed positive for parasitemia were included in the study. Animals were euthanized at 14 dpi for organ collection. In this experimental model, this time point corresponds to the period of maximal cardiac impairment, occurring after the onset of infection-induced mortality, which usually begins around 11 dpi and can extend up to 21 dpi, when complete mortality is typically observed (34). Additionally, treatment in non-infected animals was also evaluated to assess potential adverse effects of LDT409 (**Supplementary Figure 2**). The experimental design is shown in **Figure 4**.

### Parasitemia, body weight, muscle strength, and mortality

Parasitemia was assessed individually by direct microscopy using the *Pizzi-Brener* method (35). Briefly, 5 µL of blood was collected and mounted on a slide with a coverslip, and 50 random microscopic fields were examined. Body weight was measured at the beginning of treatment (5 dpi) and at 14 dpi using a precision balance. The result was expressed as percentage weight gain or loss, corresponding to the change in body weight from the start of treatment to the end of the experiment. Cumulative mortality was monitored daily, and the survival rate was calculated at 14 dpi. Muscle strength, a non-invasive indicator of the animals’ physical condition, was evaluated at 14 dpi using a grip strength meter (Grip, FEP 305, Insight, Brazil) as previously described (36). Data were expressed as mean strength intensity in gram-force (gf) per body weight (g).

### Biochemical analysis

At 14 dpi, blood was collected by cardiac puncture after euthanasia for plasma separation. Hepatic injury was evaluated by measuring alanine aminotransferase (ALT) and aspartate aminotransferase (AST), while cardiac injury was assessed by measuring creatine kinase isotype MB (CK-MB). Commercial kits were used according to the manufacturer’s instructions (LabTest Laboratory, Minas Gerais, Brazil).

### Histopathological analysis

At 14 dpi, heart and liver tissues were collected for histopathological analysis. For this procedure, five animals per group were randomly selected. Samples were embedded in Tissue-Tek OCT (Sakura, Torrance, USA), frozen in liquid nitrogen, and stored at -80 °C. Cardiac sections, 3 µm thick, were obtained using a cryostat (CM1850; Leica Biosystems, Wetzlar, Germany) and fixed in 4% paraformaldehyde solution for 30 minutes at room temperature. For histopathological analysis, the sections were stained with hematoxylin and eosin (H&E) or picrosirius red. At least five fields per sample were analyzed by light microscopy (AxioLab A1; Zeiss, Oberkochen, Germany). Quantitative evaluation included the percentage of area (i) positive for collagen and (ii) occupied by cell nuclei.

### Cytokine analysis

Plasma was collected at 14 dpi and stored at -80 °C until analysis. Protein levels of interleukin-2 (IL-2), interleukin-4 (IL-4), interleukin-6 (IL-6), interleukin-10 (IL-10), tumor necrosis factor (TNF), interferon-γ (IFN-γ), and interleukin-17A (IL-17A) were quantified in individual samples using the BD Cytometric Bead Array (CBA) Mouse Inflammation Kit (Becton, Dickinson, New Jersey, USA) according to the manufacturer’s recommendations. Data were acquired on a FACSCalibur flow cytometer (Becton, Dickinson) and analyzed with FCAP software (Becton, Dickinson).

### ECG analysis

ECG recordings were obtained from physically restrained, non-sedated mice at 14 dpi for infected animals, or 5 days after treatment initiation for non-infected mice, following previously described protocols (34). Briefly, animals were placed in the supine position, and subcutaneous electrodes were carefully positioned according to the standard lead II (DII) configuration. ECG signals were recorded and analyzed to determine P wave duration and PR, QRS, and QT interval durations, expressed in milliseconds (ms), while heart rate was calculated and expressed as beats per minute (bpm).

### Statistical analysis

For *in vitro* experiments, data represent at least three independent experiments unless otherwise stated. For *in vivo* assays, each experimental group consisted of 7 to 10 mice. Results are expressed as means and standard error of the mean (SEM) for each group. The normality of data distribution was assessed using the Shapiro–Wilk test. Parametric data were analyzed using one-way analysis of variance (ANOVA), followed by Fisher’s least significant difference (LSD) post-hoc test without correction for multiple comparisons. For non-parametric data, between-group comparisons were performed using the Kruskal–Wallis test, followed by Dunn’s post-hoc test without correction. Associations between continuous variables were evaluated using Spearman’s rank correlation analysis. All statistical analyses were conducted using GraphPad InStat version 8.0 (GraphPad Software, Inc., La Jolla, CA, USA). P values < 0.05 were considered statistically significant.

## Results

### *In silico* evaluation indicates that LDT409 meets drug-likeness and safety criteria

**Figure 1:**
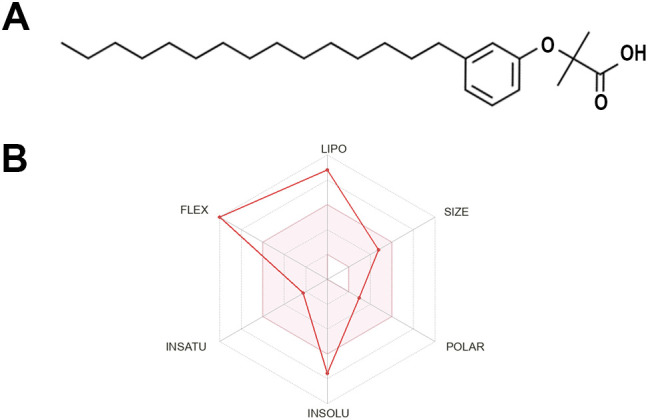
Physicochemical properties of LDT409. **(A)** Chemical structure and **(B)** Bioavailability Radar generated in SwissADME. The pink area represents the optimal range for each property: lipophilicity (XLOGP3 between −0.7 and +5.0), size (molecular weight between 150 and 500 g/mol), polarity (TPSA between 20 and 130 Å^2^), solubility (log S ≤ 6), saturation (fraction of carbons in sp^3^ hybridization ≥ 0.25), and flexibility (≤ 9 rotatable bonds).

*In silico* predictions revealed that LDT409 complies with four of Lipinski’s rules, displaying a relatively low molecular weight (MW = 390.60), fewer than five hydrogen-bond donors (HBDs < 5), fewer than ten hydrogen-bond acceptors (HBAs < 10), and a low polar surface area (PSA < 46.53 Å^2^), thus meeting most drug-likeness criteria. However, the compound falls outside the desired lipophilicity profile (LOGP _o/w_ = 7.07; MLOG > 4.15) (**Figure 1**; **Table 1**). ADMET predictions suggested that LDT409 is not permeable to the blood-brain barrier (BBB) but shows high potential for intestinal absorption (**Table 1**). Regarding drug metabolism, LDT409 did not show CYP inhibitory promiscuity. Additionally, LDT409 exhibited a low risk of hepatotoxicity, nephrotoxicity, mutagenicity (absence of Ames test induction), and carcinogenicity. Although *in silico* analysis predicted potential cardiotoxicity of LDT409 via hERG potassium channel inhibition, ECG assessment in non-infected animals revealed no alterations in P wave, PR, QRS, or QT intervals, nor in heart rate, indicating the absence of acute cardiotoxic effects associated with LDT409 administration. (**Table 1**; **Supplementary Figure 3**). Potential molecular target prediction in *Mus musculus* identified 100 proteins (**Supplementary Table 1**), most of which were classified as G protein-coupled receptors (24%) and nuclear receptors (16%). **Table 1** highlights molecules already described in the literature as potential targets of LDT409 (15).

**Table 1:** Physicochemical, ADMET properties, and *in silico* prediction of LDT409.

Physicochemical properties	
Formula	C_25_H_42_O_3_
Molecular weight	390.60 g/mol
Fraction Csp^3^	0.72
Number of rotatable bonds	17
TPSA	46.53 Å
LOGP _o/w_	7.07
LogS (ESOL)	-7.55 (Poorly soluble)
Druglikeness	
Lipinski	Yes, 1 violation: MLOG > 4.15
*In silico* prediction	
ADMET predicted profile	Value
Intestinal Absorption	+
Blood Brain Barrier	–
Oral bioavailability	–
Subcellular localization	Mitochondria
CYP2C9 substrate	+
CYP2D6 substrate	–
CYP3A4 inhibition	–
CYP2C19 inhibition	–
CYP1A2 inhibition	–
CYP inhibitory promiscuity	–
Carcinogenicity (binary)	–
Carcinogenicity (trinary)	Non-required
Ames mutagenesis	–
hERG inhibition	+
Hepatotoxicity	–
Nephrotoxicity	–
Molecular Target in *Mus musculus*	Uniprot ID
Peroxisome proliferator-activated receptor alpha	Q07869
Peroxisome proliferator-activated receptor gamma	P37231
Peroxisome proliferator-activated receptor delta	Q03181

### LDT409 displays dose-dependent trypanocidal activity against bloodstream and intracellular forms of *T. cruzi*

After *in silico* analysis, the activity of LDT409 against the BT forms of the Y strain (DTU II) was evaluated. As shown in **Table 2**, the activity of LDT409, expressed as EC_50_/24h, was approximately 300 μM. To further assess its trypanocidal potential, LDT409 was tested against intracellular forms of the Y strain using HMCs as host cells. To determine the concentrations for the trypanocidal assays, we first assessed the cytotoxicity of the compound on host cells. The LC_50_ values for HMCs were 440.19 ± 88.97 μM at 24 h and 131.31 ± 12.35 μM at 48 h. These results indicate that the compound’s cytotoxicity increases over time. Based on the LC_50_ values, a range of non-toxic concentrations between 25 and 100 μM was established for subsequent assays to evaluate the trypanocidal activity of the compound in intracellular amastigotes.

**Table 2:** Activity of LDT409 *in vitro.*.

409		
Cells	Parameters	µM
BT	EC_50_/24h	^a^ 331.31 ± 11.22
Intracellular Forms	EC_50_/24h	^b^ 42.09 ± 2.62
	EC_50_/48h	^b^ 48.28 ± 22.30
Cardiac Cell (HMCs)	LC_50_/24h	^c^ 440.19 ± 88.97
	LC_50_/48h	^c^ 131.31 ± 12.35
	SI/24h	^d^ 10.45
	SI/48h	^d^ 2.71

Mean ± standard deviation of three independent experiments.

a Treatment for 24 h at 37 °C of the trypomastigotes of *T. cruzi*.

b Treatment for 24 h or 48 h at 37 °C of the intracellular amastigotes of *T. cruzi*.

c Treatment for 24 h or 48 h at 37 °C of non-infected HMCs.

d Selectivity index (SI) = LC_50_ of HMCs/EC_50_ of intracellular amastigotes.

**Figure 2:**
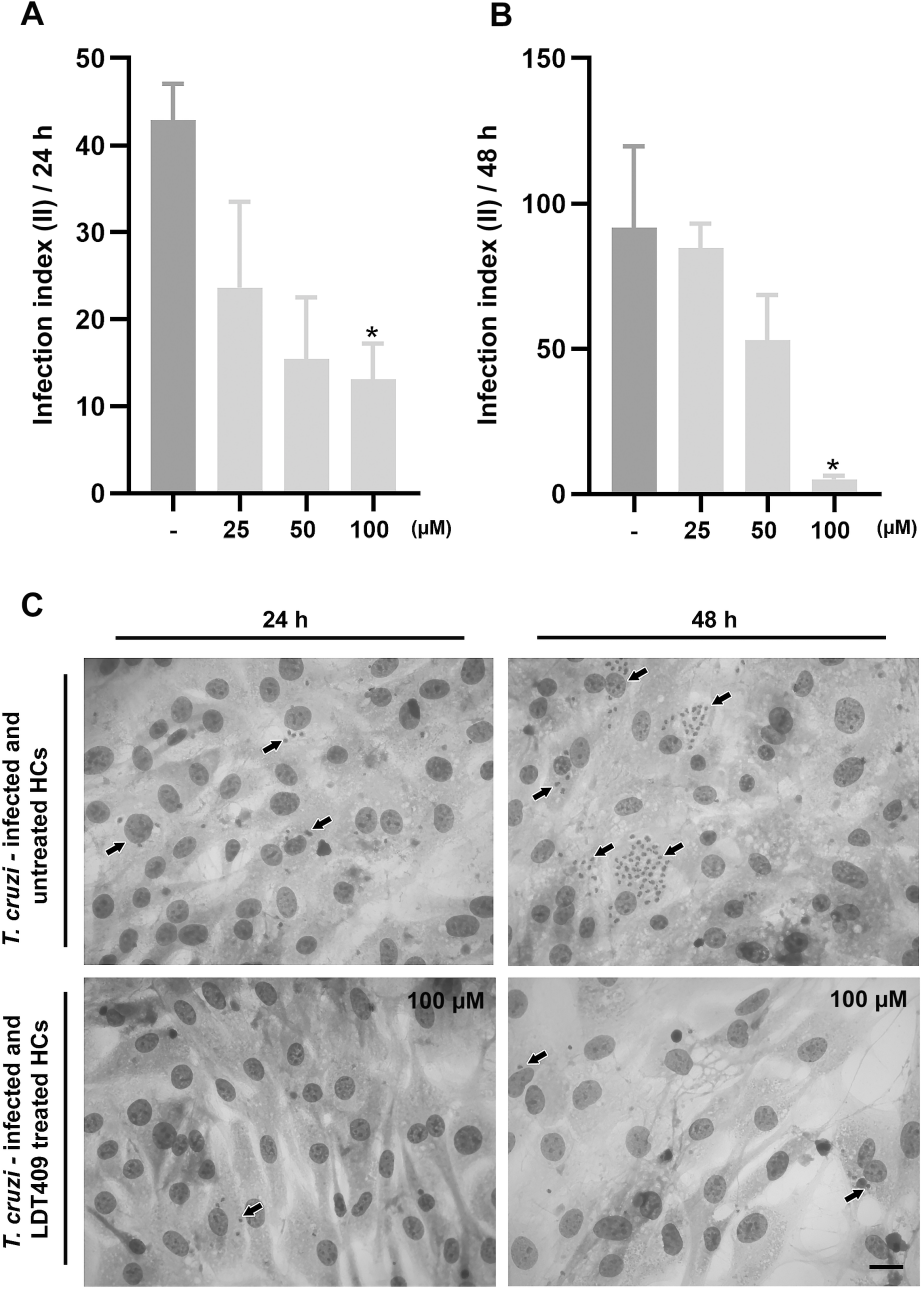
Effects of LDT409 on *T. cruzi* (Y strain)-infected HMCs. Graphical representation of the dose-dependent inhibition of the infection index at **(A)** 24 h and **(B)** 48 h post-treatment. **(C)** Representative photomicrographs of *T. cruzi*-infected HMCs, treated (100 μM) or not with LDT409, fixed and stained with Diff-Quick. Black arrows correspond to intracellular forms of the parasite (Bar = 20 μm); Data were analyzed by one-way ANOVA followed by Fisher’s LSD post-hoc test. *Different from untreated control group. *p < 0.05.

Quantification of infected and treated cultures demonstrated dose-dependent inhibition of infection (**Figures 2A, B**). Light microscopy showed that treatment with 100 µM LDT409 for 24 and 48 h inhibited HMCs infection by *T. cruzi* (**Figure 2C**). In addition, the EC_50_ for intracellular amastigotes was calculated at 24 and 48 h to determine the selectivity index (SI = LC_50_/EC_50_), which was 10.45 and 2.71, respectively. These results indicate that although the compound’s cytotoxicity increases over time, its trypanocidal activity remains largely unchanged, resulting in reduced selectivity with prolonged exposure.

### LDT409 induces ultrastructural alterations, particularly affecting mitochondria, in *T. cruzi* trypomastigotes

**Figure 3:**
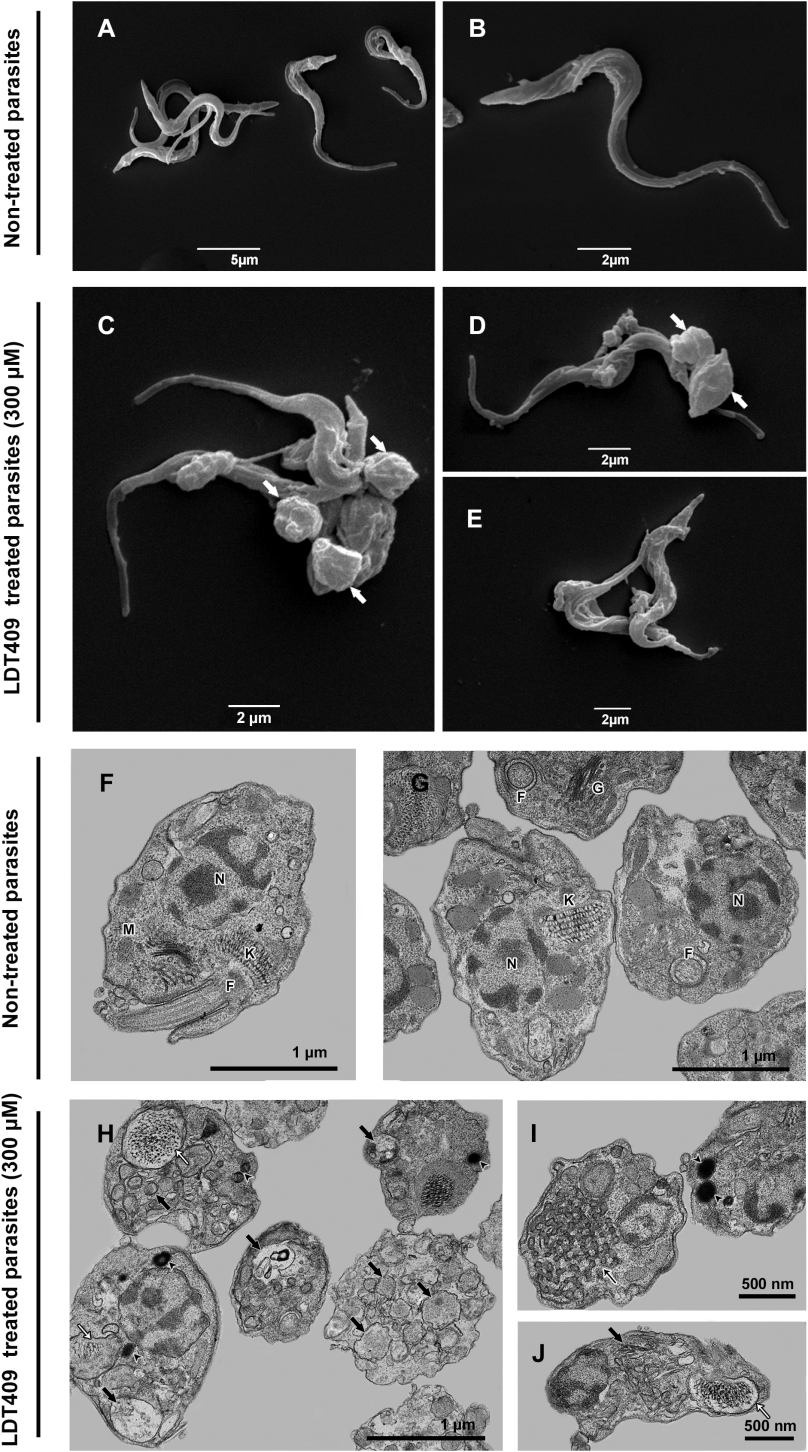
Ultrastructural alterations in *T. cruzi* trypomastigote forms induced by LDT409. Representative photomicrographs of trypomastigote forms analyzed by SEM **(A, B)** non-treated parasites (control); **(C-E)** LDT409-treated parasites (IC_50_/24h = 300 μM) and TEM **(F, G)** non-treated parasites; **(H-J)** LDT409-treated parasites. White arrow shows a rounding of the parasite’s body and alterations, such as surface shrinkage and flagellum shortening; Thin white arrow shows changes in mitochondrial and kinetoplast morphology; Black arrow shows cytoplasmic vacuoles, including the accumulation of lipid bodies. N: nucleus; M: mitochondria; K: kinetoplast; F: flagellum; G: Golgi.

To assess the effect of LDT409 on the parasite, ultrastructural alterations in *T. cruzi* trypomastigotes were examined by electron microscopy at a concentration corresponding to its IC_50_/24h value (300 μM). Compared with untreated cells, SEM analysis qualitatively revealed that LDT409 induced morphological changes in trypomastigotes (**Figure 3**), including flagellum shortening, rounding of the parasite’s body, and surface shrinkage (**Figures 3C–E**). TEM was also used to evaluate the ultrastructural phenotype of parasites. We observed the formation of cytoplasmic vacuoles, accumulation of lipid bodies, and changes in mitochondrial and kinetoplast morphology, including kDNA compaction patterns (**Figures 3H–J**).

### LDT409 reduces the peak of parasitemia and favors the survival of infected animals

**Figure 4:**
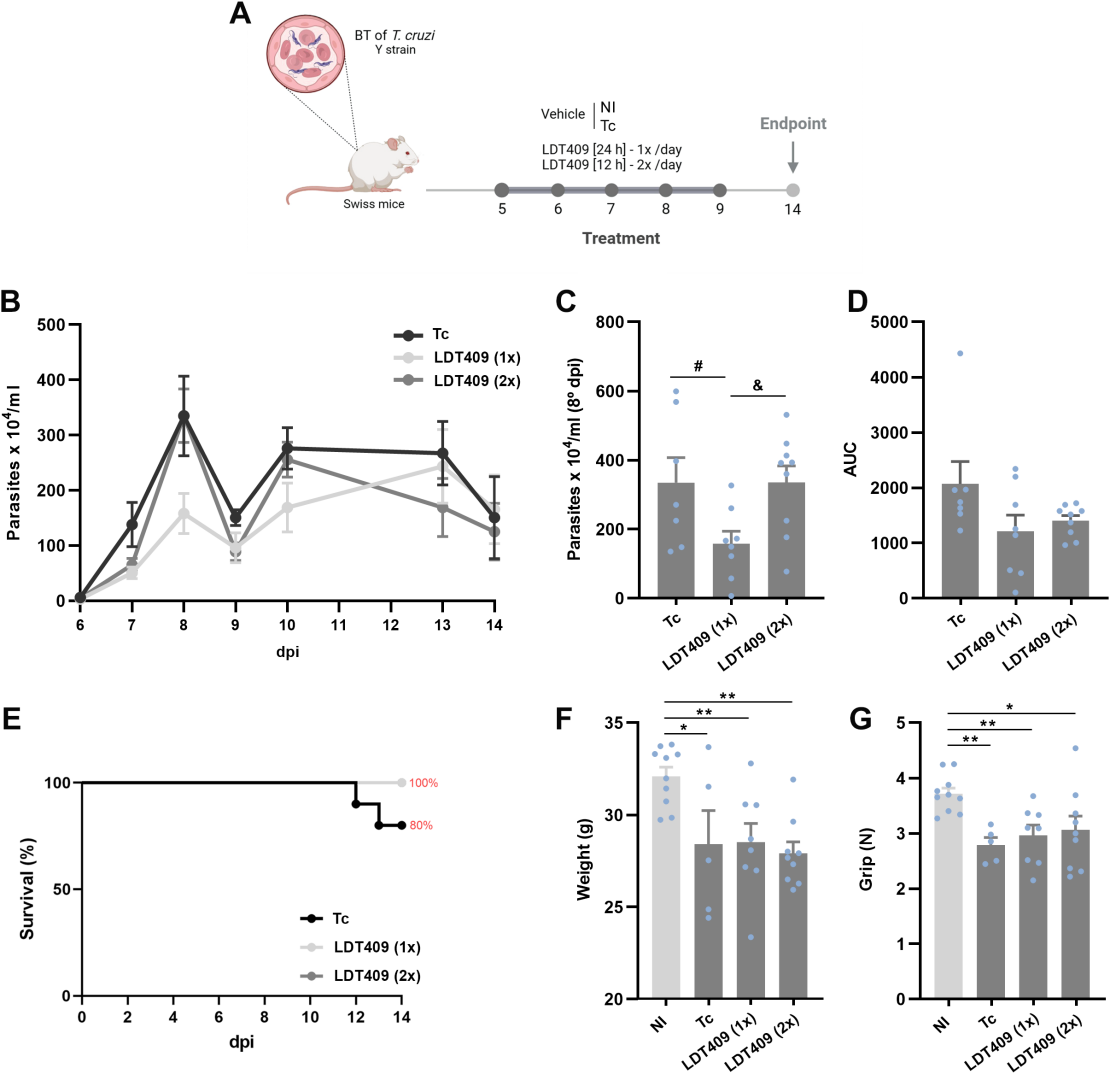
Administration of LDT409 reduces the peak of parasitemia and favors survival in *T. cruzi*-infected mice, without affecting body weight or muscle strength. **(A)** Experimental design. Male Swiss mice infected with *T. cruzi* were subjected to different treatment regimens administered by daily gavage from 5 to 9 dpi. The experimental groups included: vehicle-treated controls (NI: non-infected and Tc: *T. cruzi* infected), LDT409 at 100 mg/kg once daily (LDT409_1x_) and LDT409 at 100 mg/kg twice daily (LDT409_2x_); **(B)** parasitemia curve; **(C)** parasitemia peak (8 dpi); **(D)** area under the curve **(AUC)** of parasitemia (5 to 14 dpi); **(E)** survival rate (%); **(F)** body weight (g) and **(G)** muscular strength (N) at 14 dpi. Parametric data were analyzed by one-way ANOVA followed by Fisher’s LSD post-hoc test, while non-parametric data were analyzed using the Kruskal–Wallis test followed by Dunn’s post-hoc test. ^#^Different from Tc; *different from NI; ^&^different from LDT409_1x._ * ^# &^P < 0.05; **P < 0.01.

Next, we examined the effect of LDT409 on the parasitemia curve of *T. cruzi*-infected mice (**Figure 4**). A significant reduction in the parasitemia peak was observed only in the group treated once daily compared with positive-control infected mice (Tc *versus* LDT409_1x_) (**Figures 4B, C**). However, in the area under the curve (AUC) analysis, neither treatment regimen significantly reduced parasitemia (**Figure 4D**; LDT409_1x_: 1214 and LDT409_2x_: 1403 *versus* Tc: 2069; AUC of parasitemia, P>0.05). The survival curves showed that in the Tc group, mortality began at 12 dpi. The percentages of survival at 14 dpi were 100%, 100%, and 80% for LDT409_1x_, LDT409_2x,_ and Tc, respectively (**Figure 4E**). At this time point, both treatment regimens failed to prevent the decline in body weight and muscle strength caused by infection, relative to uninfected mice (negative-control group; NI) (**Figures 4F, G**).

### LDT409 preserves hepatic integrity but induces renal alterations in *T. cruzi* infection

**Figure 5:**
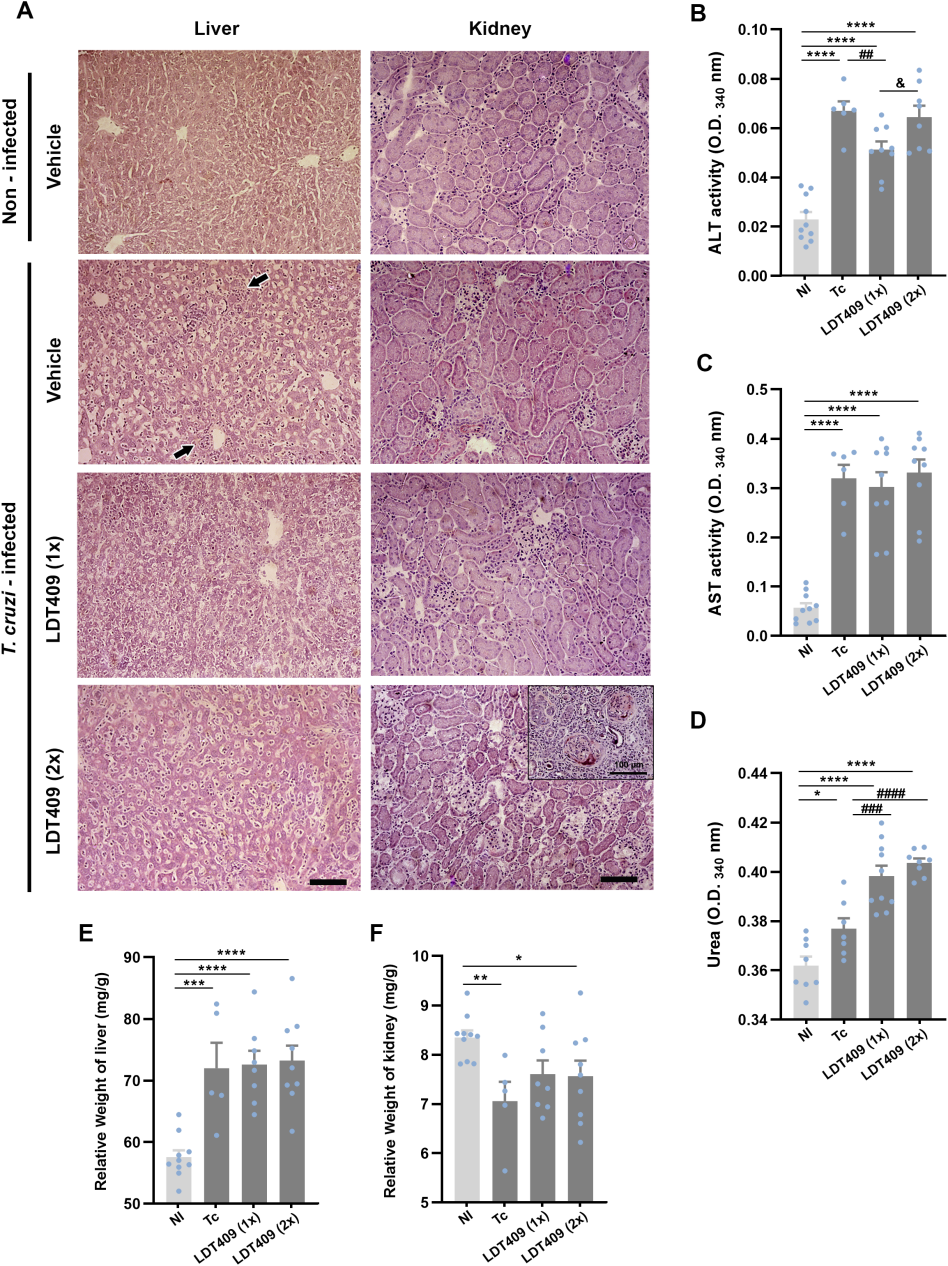
Administration of LDT409 (once or twice daily) does not exacerbate hepatic damage but may cause renal impairment in *T. cruzi*-infected mice. **(A)** Representative photomicrographs of liver and kidney tissues from *T. cruzi*-infected mice stained with H&E; the inset shows morphological changes compatible with AIN and intraglomerular fibrin deposition. Plasma activity of **(B)** ALT; **(C)** AST; **(D)** urea. Relative height of **(E)** liver and **(F)** kidney (mg/g). Black arrow shows an inflammatory infiltrate. Bar = 500 µm. Parametric data were analyzed by one-way ANOVA followed by Fisher’s LSD post-hoc test, while non-parametric data were analyzed using the Kruskal–Wallis test followed by Dunn’s post-hoc test. ^#^Different from Tc; *different from NI; ^&^ different from LDT409_1x._* ^&^P < 0.05; ** ^##^P < 0.01; *** ^###^P < 0.001; **** ^####^P < 0.0001.

Qualitatively, histopathological analysis of hepatic tissue showed high numbers of inflammatory cells and sinusoidal dilatation in both untreated (Tc) and treated mice (LDT409_1x_ and LDT409_2x_), compared with NI (**Figure 5A**). All infected groups also showed a significant increase in ALT activity (**Figure 5B**; NI: 0.02 *versus* Tc: 0.06; LDT409_1x_: 0.05 and LDT409_2x_: 0.06; optical density at 340 nm [OD_340_], *P* < 0.05), AST activity (**Figure 5C**; NI: 0.05 *versus* Tc: 0.31; LDT409_1x_: 0.30 and LDT409_2x_: 0.33; OD_340_, *P* < 0.001), and an increase in relative liver weight (**Figure 5E**; NI: 57.56 *versus* Tc: 72.01; LDT409_1x_: 72.58 and LDT409_2x_: 73.24; mg/g, *P* < 0.01).

In the qualitative histological analysis of kidney sections, no alterations resulting from the infection were observed (NI *versus* Tc *versus* LDT409_1x_) (**Figure 5A**). However, in the LDT409_2x_ treatment group, we observed acute interstitial nephritis (AIN) and intraglomerular fibrin deposition, as shown in the inset (**Figure 5A**). Consistent with these morphological findings, LDT409_2x_ mice exhibited increased serum urea levels compared with Tc, suggesting potential nephron damage associated with the administration of LDT409 twice daily (**Figure 5D**; Tc: 0.37 *versus* LDT409_2x_: 0.40; OD_340,_ *P* < 0.05). No significant changes in relative kidney weight were observed among infected groups (**Figure 5F**; Tc: 7.06 *versus* LDT409_1x_: 7.60; LDT409_2x_: 7.56; mg/g, *P* > 0.05).

### LDT409 mitigates cardiac injury in *T. cruzi*-infected mice

**Figure 6:**
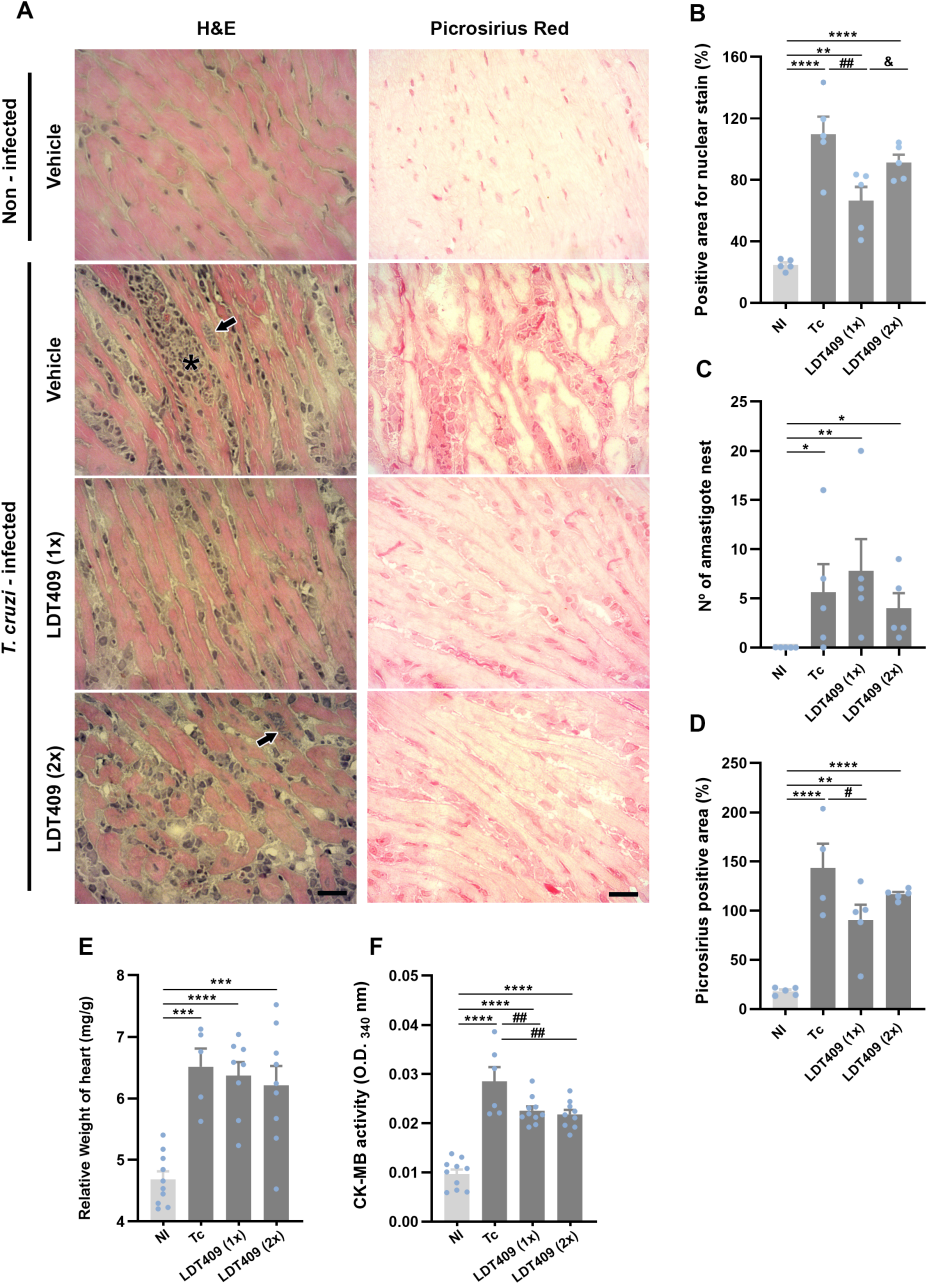
Administration of LDT409 prevents cardiac damage in *T. cruzi*-infected mice. **(A)** Representative photomicrographs of cardiac tissue from *T. cruzi* infected mice stained with H&E and picrosirius red; **(B)** Percentage area occupied by cell nuclei (hematoxylin); **(C)** Number of parasite nests in cardiac tissue; **(D)** Quantification of collagen-positive area (red staining); **(E)** Relative heart height (mg/g) and **(F)** Activity of plasmatic CK-MB. Asterisk shows an inflammatory infiltrate and black arrows show parasite nests. Bar = 500 µm. Parametric data were analyzed by one-way ANOVA followed by Fisher’s LSD post-hoc test, while non-parametric data were analyzed using the Kruskal–Wallis test followed by Dunn’s post-hoc test. #Different from Tc; *different from NI; &different from LDT4091x. ^* # &^ P < 0.05; ^** ##^ P < 0.01; *** P < 0.001; ****P < 0.0001.

Myocardial sections from the Tc group showed numerous diffusely distributed inflammatory foci, often associated with necrotic areas, amastigote nests, loss of cardiac fiber integrity, and collagen deposition (**Figure 6**). A reduction in the number of stained cell nuclei (hematoxylin) was observed only in the group treated once daily compared with Tc (**Figures 6A, B**; Tc: 109.6 *versus* LDT409_1x_: 66.48; positive area [%], P<0.0001). Neither treatment regimen reduced the number of amastigote nests (**Figure 6C**). Moreover, compared to Tc, collagen deposition was significantly reduced in the LDT409_1x_ group (**Figure 6D**; Tc: 143.6 *versus* LDT409_1x_: 90.19; positive area [%], P = 0.01). Due to technical limitations, reliable ECG recordings could only be obtained for the LDT409_1x_ group. Despite these structural and inflammatory improvements, LDT409_1x_ treatment did not ameliorate the electrical alterations induced by *T. cruzi* infection (**Supplementary Figure 4**). Additionally, relative heart weight was not reduced in either therapeutic scheme with LDT409, compared with Tc (**Figure 6E**; Tc: 6.40 *versus* LDT409_1x_: 6.09; mg/g, P = 0.0003). However, plasma creatine kinase isotype MB (CK-MB) activity was lower in both the LDT409_1x_ and LDT409_2x_ groups (**Figure 6F**; Tc: 0.03 *versus* LDT409_1x_: 0.02 and LDT409_2x_: 0.02; OD_340_, P <0.05).

### LDT409 modulates the inflammatory response in *T. cruzi*-infected mice

**Figure 7:**
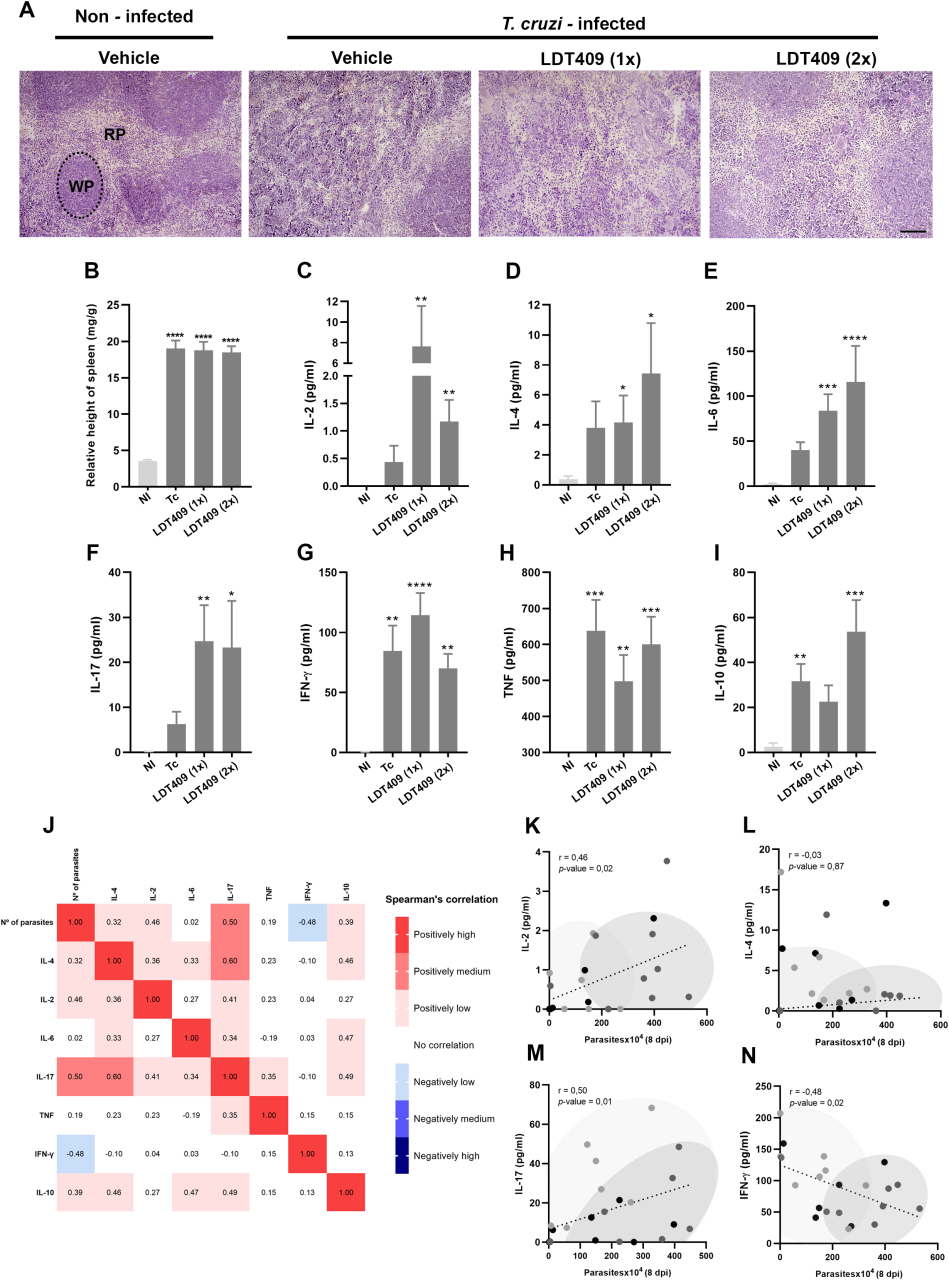
Administration of LDT409 modulates the immune response in *T. cruzi*-infected mice. **(A)** Representative photomicrograph of spleen tissue stained with H&E, showing white pulp (WP) and red pulp (RP); **(B)** relative spleen weight (mg/g); Levels of cytokine in plasma of **(C)** IL-2; **(D)** IL-4 **(E)** IL-6 **(F)** IL-17 **(G)** IFN-γ **(H)** TNF **(I)** IL-10; **(J)** Spearman’s correlation matrix; Spearman’s correlation of parasitemia and **(K)** IL-2; **(L)** IL-4; **(M)** IL-17; **(N)** IFN-γ; Black dots represent Tc; light gray dots represent LDT409_1x_ and dark gray dots represent LDT409_2x_. Parametric data were analyzed by one-way ANOVA followed by Fisher’s LSD post-hoc test, while non-parametric data were analyzed using the Kruskal–Wallis test followed by Dunn’s post-hoc test. *different from NI; *P < 0.05; **P < 0.01; ***P < 0.001; ****P < 0.0001.

As expected, *T. cruzi* infection was associated with splenomegaly and structural disorganization of the spleen, making it difficult to distinguish between the white pulp (WP) and red pulp (RP) (**Figure 7A**). This qualitative observation indicates that LDT409 treatment did not appear to reduce this structural disorganization (**Figure 7A**). Moreover, the treatment did not decrease the splenomegaly induced by the infection (**Figure 7B**; Tc: 20.65 *versus* LDT409_1x_: 20.01 and LDT409_2x_: 18.27 mg/g, P > 0.05). At 14 dpi, administration of LDT409 induced the production of IL-2, IL-4, IL-6, and IL-17 (NI *versus* LDT409_1x_ and LDT409_2x_; pg/ml, P < 0.05), which was not observed in Tc (NI *versus* Tc; P > 0.05) (**Figures 7C–F**). In addition, all *T. cruzi*-infected mice exhibited increased plasma levels of IFN- γ, TNF, and IL-10 compared with NI (**Figures 7G–I**).

To further investigate the relationship between cytokine production and parasitemia control, we performed a Spearman’s correlation matrix and classified associations between variable pairs according to the correlation coefficients (37) (**Figure 7J**). Parasitemia levels were positively associated with IL-2, IL-4, IL-17, and IL-10 and negatively associated with IFN-γ levels in plasma (**Figure 7J**). To better visualize these associations, each pair of correlated variables was analyzed separately, allowing the distribution of individual mice to be observed (**Figures 7K–N**). Mice in the LDT409_2x_ group clustered together (dark gray area), presenting higher levels of IL-2, IL-4, IL-17, and lower levels of IFN-γ (**Figures 7K–N**). Finally, we calculated the ratio between IL-10 and IFN-γ in infected experimental groups (Tc: 0.34, LDT409_1x_: 0.23, and LDT409_2x_: 0.74, pg/ml). In LDT409_2x_ this ratio was significantly higher than in LDT409_1x_ (LDT409_1x_ *versus* LDT409_2x_ IL-10/IFN- γ, P = 0.009).

## Discussion

Chagas disease (CD) remains a significant public health concern, affecting neglected populations, primarily in tropical and subtropical regions. Due to limited interest from most pharmaceutical companies and the complexity of the disease, CD still lacks a vaccine and safer, more effective treatment alternatives (38). In this context, our group has been engaged for several years in investigating the effects of bioactive compounds on *T. cruzi*, using natural or semisynthetic samples with low production costs (39–42).

Following this approach, in recent years, our group has initiated a drug discovery project aimed at developing new molecules that meet sustainability requirements. By adopting the emerging *waste-to-pharma* concept, we have explored the potential of developing new bioactive compounds from cashew nut shell liquid (CNSL), an inexpensive and widely available byproduct of the cashew industry (12). This strategy is considered promising, particularly for the development of drugs targeting neglected tropical diseases (12, 17, 43).

Nunes Lemes et al. (2023) evaluated the activity of six phospholipid derivatives obtained by hemisynthesis from anacardic acid, cardanol, and cardol extracted from CNSL (17). These compounds were tested for their antiparasitic activity *in vitro* against different evolutive forms of *T. cruzi* of the Y strain (epimastigotes, trypomastigotes, and amastigotes). Four showed greater activity and selectivity against *T. cruzi* compared with Bz, and were classified as hit compounds according to the criteria established by the Drugs for Neglected Diseases initiative (DNDi) for CD (38, 44).

These results prompted our group to investigate the trypanocidal activity of LDT409, another CNSL derivative. We first evaluated its *in vitro* activity against the two infective forms of the parasite (trypomastigotes and amastigotes). LDT409 reduced the infection index in *T. cruzi*-infected HMCs and eliminated trypomastigote forms, inducing ultrastructural alterations, particularly in the mitochondria and kinetoplast. These qualitative observations are consistent with *in silico* analyses indicating that LDT409 preferentially accumulates in mitochondria. Although its potency against trypomastigotes (LDT409 IC_50_/24 h = 331.31 ± 11.22 µM) was lower than that of the reference drug benznidazole (Bz IC_50_/24 h = 8.82 ± 1.08 µM) (39), LDT409 produced biologically relevant effects, including a potential anti-inflammatory profile, supporting its further investigation as a multifunctional candidate.

We next assessed the *in vivo* trypanocidal activity of LDT409. Treatment with this compound at a dose of 100 mg/kg once daily (LDT409_1x_) reduced the parasitemia peak at 8° dpi, whereas no significant changes were detected in the number of cardiac amastigote nests. This apparent dissociation may be related to the physicochemical properties of LDT409, notably its high lipophilicity, which could favor preferential distribution to lipid-rich tissues such as the liver and adipose tissue (45, 46). Circulating trypomastigotes are directly exposed to the compound during periods of systemic availability, which may be sufficient to impact parasitemia even in the context of progressive tissue redistribution. In contrast, activity against intracellular cardiac amastigotes likely requires sustained myocardial exposure and intracellular drug accumulation, which may be limited under these conditions (47). Thus, tissue-specific distribution and pharmacokinetic constraints may contribute to differential antiparasitic effects in circulation compared with the myocardium.

Moreover, contrary to our expectations, treatment administered twice daily (LDT409_2x_) did not reduce parasitemia. Our initial hypothesis was that oral administration every 12 h – approximately matching the plasma clearance time – would improve parasite elimination by maintaining higher plasma drug concentrations, while the increased cumulative dose (200 mg/kg/day) could further enhance this effect (15). However, this was not observed, suggesting that the unexpected outcome may result from complex pharmacokinetic or host–parasite interactions, possibly influenced by the immunomodulatory action of LDT409.

**Figure 8:**
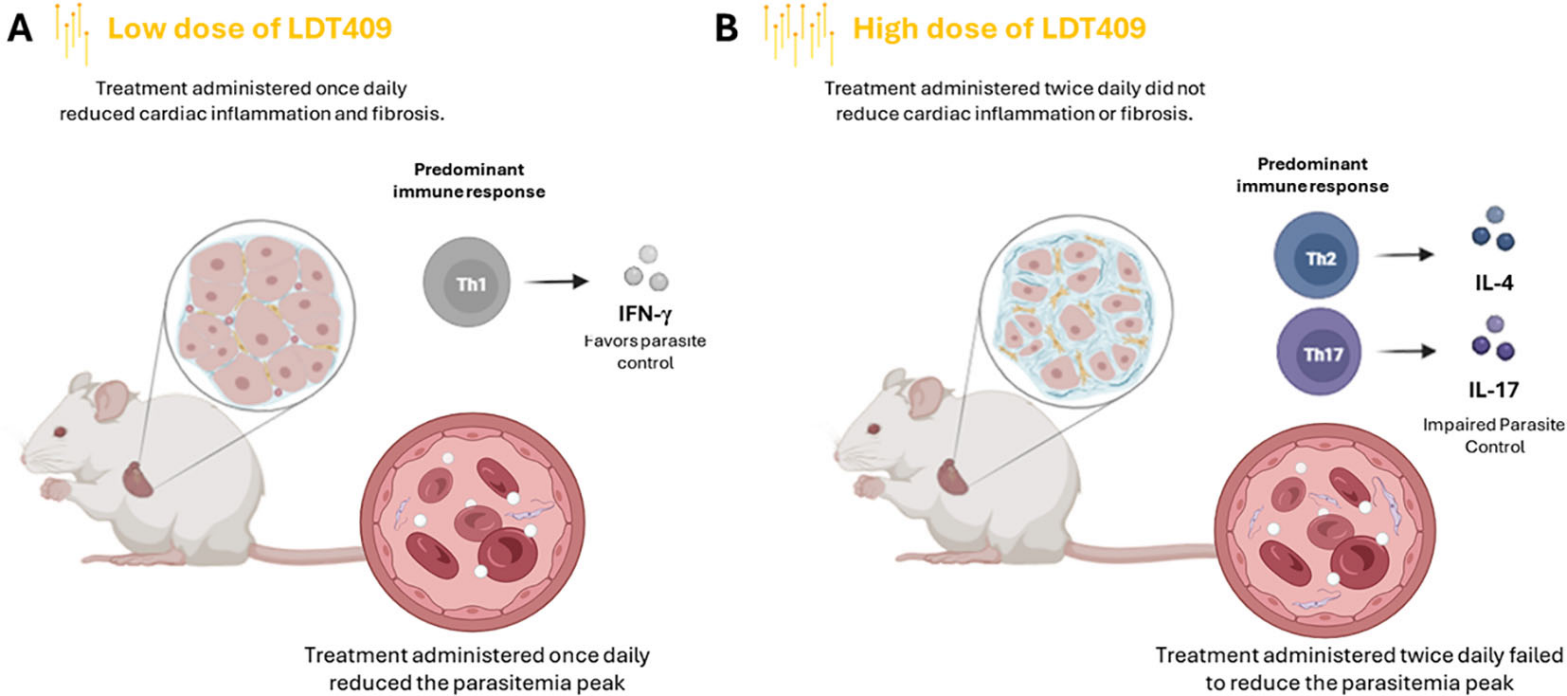
Proposed immunomodulatory effects of LDT409 during acute *Trypanosoma cruzi* infection. **(A)** Treatment with LDT409 at lower cumulative doses exerts a dual effect by combining its trypanocidal activity with a controlled maintenance of the Th1 response, resulting in effective parasite control and attenuation of deleterious cardiac effects. **(B)** In contrast, treatment with LDT409 at higher cumulative doses favors polarization toward Th2/Th17 immune responses, overriding the trypanocidal potential of the drug and impairing infection control. We hypothesize that this effect is mediated by the agonistic action of LDT409 on PPAR receptors, leading to suppression of NF-κB–dependent inflammatory pathways; however, this mechanism still needs to be fully elucidated.

Analysis of variable associations revealed that treatment with LDT409 modulated the immune response against the parasite, as the treated groups showed higher levels of IL-2, IL-4, and IL-17 and an increased IL-10/IFN-γ ratio. This immunological effect was most evident in the group treated with the highest cumulative dose (LDT409_2x_). In this experimental group, the promotion of a Th2- and Th17-type immune response may have counteracted the trypanocidal effect of LDT409 (48). Based on these results, we hypothesize that treatment with LDT409, in this mouse model, acts as both a trypanocidal agent and an immunomodulator, favoring a Th2/Th17-type immune response to the detriment of a Th1 pro-inflammatory response (**Figure 8**).

We observed that *in vivo* treatment with LDT409 once daily (LDT409_1×_) reduced peak parasitemia, mortality, cardiac inflammation, fibrosis, and cardiac damage, as indicated by CK-MB activity. In contrast, treatment with the higher cumulative dose (LDT409_2×_) did not produce significant changes in these parameters. Additionally, treatment administered twice daily increased plasma urea concentrations, suggesting potential renal damage associated with this regimen (49). Thus, our findings indicate that at higher doses (200 mg/kg/day), LDT409 impairs the host immune response’s ability to control infection, thereby diminishing the drug’s trypanocidal efficacy. Conversely, at lower doses (100 mg/kg/day), LDT409 attenuates myocarditis while preserving the immune system’s capacity to control parasitism, without causing undesirable renal side effects.

Although LDT409 exhibits trypanocidal activity, this compound was originally developed as a ligand for PPARs and can modulate multiple metabolic and inflammatory pathways that play a critical role in the pathophysiology of CD. Its ability to act as a partial agonist of PPARα and PPARγ has been demonstrated both *in vitro* and *in vivo*, including studies in zebrafish experimental models and in murine models of metabolic disorders (15, 18, 21). This property was further confirmed by *in silico* analysis using a potential molecular target prediction in *Mus musculus*. Based on these findings, we hypothesized that LDT409 may exert beneficial effects in acute Chagas infection through PPAR modulation.

From a functional perspective, PPARs inhibit the production of pro-inflammatory cytokines primarily by antagonizing the activity of key transcription factors such as AP-1 and NF-κB (50). In the heart, PPAR agonists have shown efficacy as anti-inflammatory mediators (51). Notably, the effects observed with LDT409 resemble those reported for the natural PPARγ ligand 15-Deoxy-Δ12,14-Prostaglandin J2 (15dPGJ2), a well-characterized anti-inflammatory mediator. This similarity supports the idea that LDT409 may act, at least in part, as a PPAR agonist. The 15dPGJ2 is a potent anti-inflammatory molecule that represses certain genes in cardiomyocytes and activated macrophages, including NOS_2,_ cyclooxygenase 2 (COX2), and TNF-α (23, 52).

During the acute phase of *T. cruzi* infection, treatment with 15dPGJ2 reduced the inflammatory infiltrates in skeletal muscle (the site of inoculation) and decreased the number of circulating leukocytes (16). In the heart, 15dPGJ2 treatment also inhibited the expression and activity of NOS_2_, suppressed metalloproteinases 2 (MMP2) and 9 (MMP9), and reduced mRNA levels of TNF-α and IL-6 (16). However, 15dPGJ2 treatment increased parasitemia levels and cardiac parasitism, which is related to the upregulation of IL-10 levels (16).

The immunological control of *T. cruzi* involves antibodies, CD8^+^ cytotoxic T lymphocytes, and CD4^+^ Th1 helper T cells, which are characterized by the production of high levels of IFN-γ (53). Experimental studies in acute infection models have shown that pro-inflammatory cytokines such as IL-2, IFN-γ, TNF, and IL-6 are essential for resistance to the parasite. However, acute myocarditis in CD has also been associated with an exacerbated Th1 polarization relative to Th2, highlighting the dual role of these immune mechanisms (48).

In contrast, a predominant Th2 profile has been linked to increased susceptibility, with IL-4 as the main cytokine driving this response (54). Beyond the classical Th1/Th2 dichotomy, Th17 lymphocytes have emerged as additional modulators, contributing to parasite control and regulation of cardiac inflammation in experimental models, partly through modulation of Th1 responses (55). Supporting this concept, studies in mice have shown that IL-17 deficiency during the acute phase increases susceptibility to infection, accompanied by reduced expression of IFN-γ, IL-6, and TNF (56).

Our results support a model of dose- and schedule-dependent immunomodulation, potentially mediated by partial PPAR activation. Treatment with lower doses of LDT409 appears to preserve a Th1-type immune response, promoting parasite control while limiting cardiac inflammation. In contrast, higher cumulative doses shift the immune profile toward Th2/Th17 polarization, with a corresponding reduction in pro-inflammatory Th1 responses that may override the direct trypanocidal effect of the drug. The role of PPARs in mediating these effects remains a hypothesis that requires confirmation in future studies. Overall, these findings suggest that LDT409, an inexpensive and widely available byproduct, may function as an immunometabolic modulator, capable of fine-tuning host immune responses during acute *T. cruzi* infection and may represent a translationally relevant therapeutic candidate for the treatment of CD.

## Data Availability

The original contributions presented in the study are included in the article/**Supplementary Material**. Further inquiries can be directed to the corresponding author.

## Supplementary material

Supplementary Figure 1 Experimental workflow for evaluating the activity of LDT409 against intracellular *T. cruzi* amastigotes in HMCs cultures. HMCs obtained from 18-day-old mouse embryos were infected with BT and after 24 h, washed to remove noninternalized parasites. Cultures were then incubated for an additional 24 or 48 h in the absence or presence of LDT409 at serially diluted. Following treatment, cells were fixed and stained with Diff-Quick for microscopic evaluation. Antiparasitic activity was expressed as the Infection Index (II).

Supplementary Figure 2 Impact of LDT409 treatment non-infected animals. (A) body weight and (B) muscle strength in non-infected animals (C) Relative heart height (mg/g); (D) Activity of plasmatic CK-MB; (E) Relative spleen height (mg/g); (F) Relative liver height (mg/g); (G) Activity of plasmatic ALT; (H) Activity of plasmatic AST; (I) Relative kidney height (mg/g) and (J) Plasmatic urea levels. Parametric data were analyzed by one-way ANOVA followed by Fisher’s LSD *post hoc* test, while nonparametric data were analyzed using the Kruskal–Wallis test followed by Dunn’s *post hoc* test. *, Different from NI. *, P < 0.05; **, P < 0.01; ***, P < 0.001.

Supplementary Figure 3 Electrocardiographic analysis of non-infected animals treated with LDT409. (A) P wave (ms); intervals: (B) PR (ms); (C) QRS (ms); (D) QT (ms) and (E) heart rate, expressed as beats per minute (bpm). Parametric data were analyzed by one-way ANOVA followed by Fisher’s LSD *post hoc* test, while nonparametric data were analyzed using the Kruskal–Wallis test followed by Dunn’s *post hoc* test.

Supplementary Figure 4 Electrocardiographic analysis of *T. cruzi*-infected animals treated with LDT409_1x_. Intervals: (**A**) PR (ms); (**B**) QRS (ms); and (**C**) heart rate, expressed as beats per minute (bpm). Parametric data were analyzed by one-way ANOVA followed by Fisher’s LSD *post hoc* test, while nonparametric data were analyzed using the Kruskal–Wallis test followed by Dunn’s *post hoc* test. *, Different from NI. ****, P < 0.0001.
