# Supplementary figures and images for: Evaluation of the trypanocidal and immunomodulatory effects of LDT409, a cardanol derivative from cashew nut shell liquid
Source: Front Immunol. 2026 Mar 18;17:1749250. doi: 10.3389/fimmu.2026.1749250 (PMC13038549; doi:10.3389/fimmu.2026.1749250)

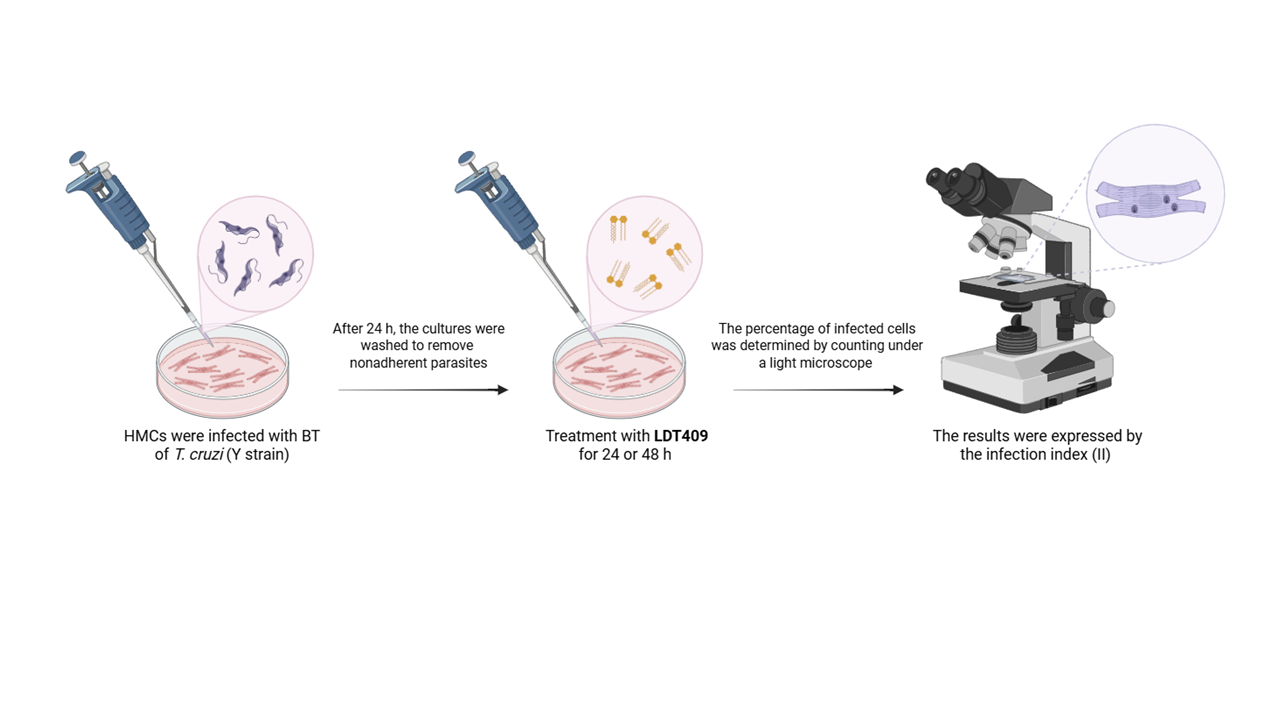

Supplement: Supplementary Figure 1 — Experimental workflow for evaluating the activity of LDT409 against intracellular T. cruzi amastigotes in HMCs cultures. HMCs obtained from 18-day-old mouse embryos were infected with BT and after 24 h, washed to remove noninternalized parasites. Cultures were then incubated for an additional 24 or 48 h in the absence or presence of LDT409 at serially diluted. Following treatment, cells were fixed and stained with Diff-Quick for microscopic evaluation. Antiparasitic activity was expressed as the Infection Index (II). [file Image1.tif]

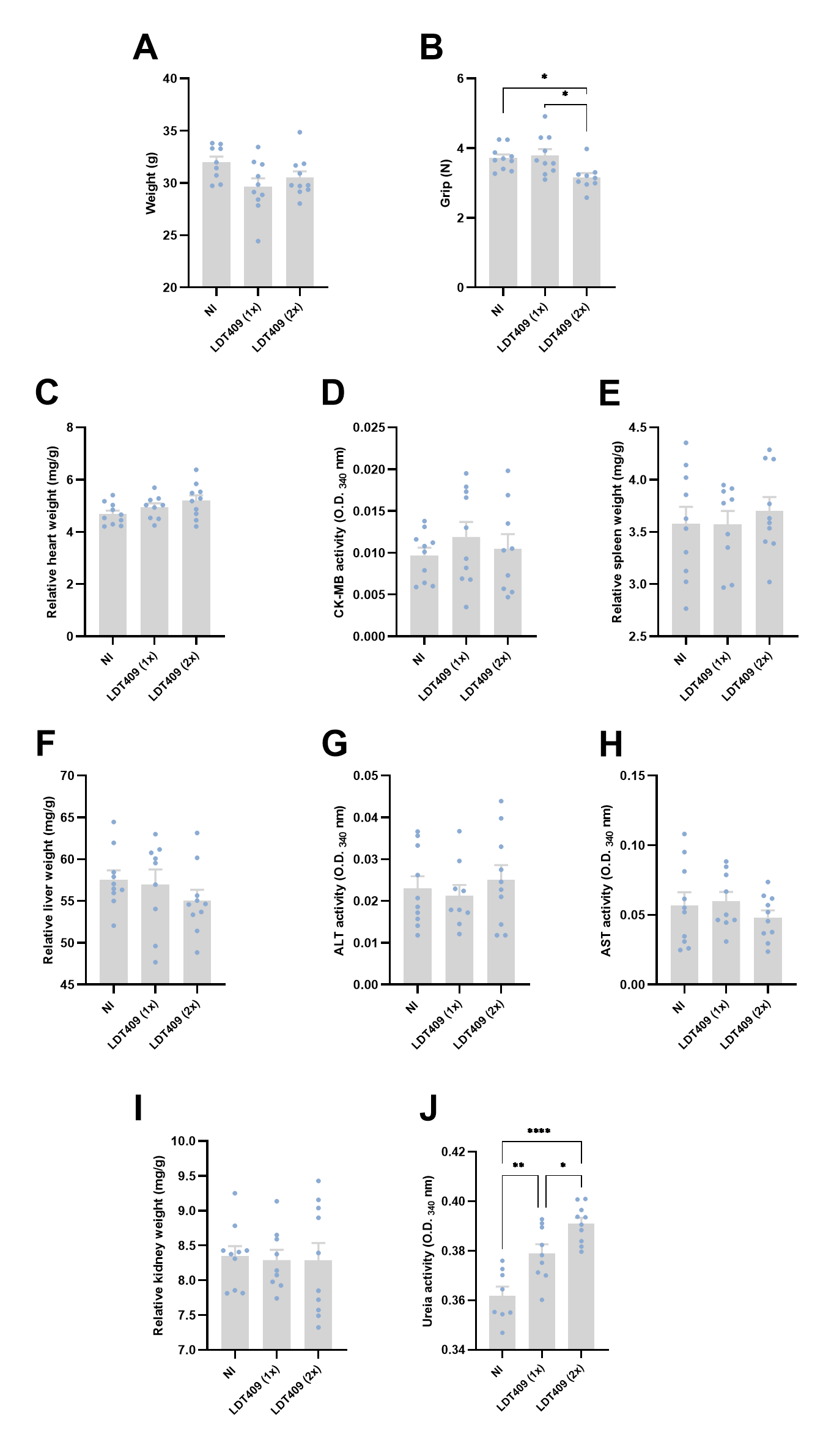

Supplement: Supplementary Figure 2 — Impact of LDT409 treatment non-infected animals. (A) body weight and (B) muscle strength in non-infected animals (C) Relative heart height (mg/g); (D) Activity of plasmatic CK-MB; (E) Relative spleen height (mg/g); (F) Relative liver height (mg/g); (G) Activity of plasmatic ALT; (H) Activity of plasmatic AST; (I) Relative kidney height (mg/g) and (J) Plasmatic urea levels. Parametric data were analyzed by one-way ANOVA followed by Fisher’s LSD post hoc test, while nonparametric data were analyzed using the Kruskal–Wallis test followed by Dunn’s post hoc test. *, Different from NI. *, P < 0.05; **, P < 0.01; ***, P < 0.001. [file Image2.tif]

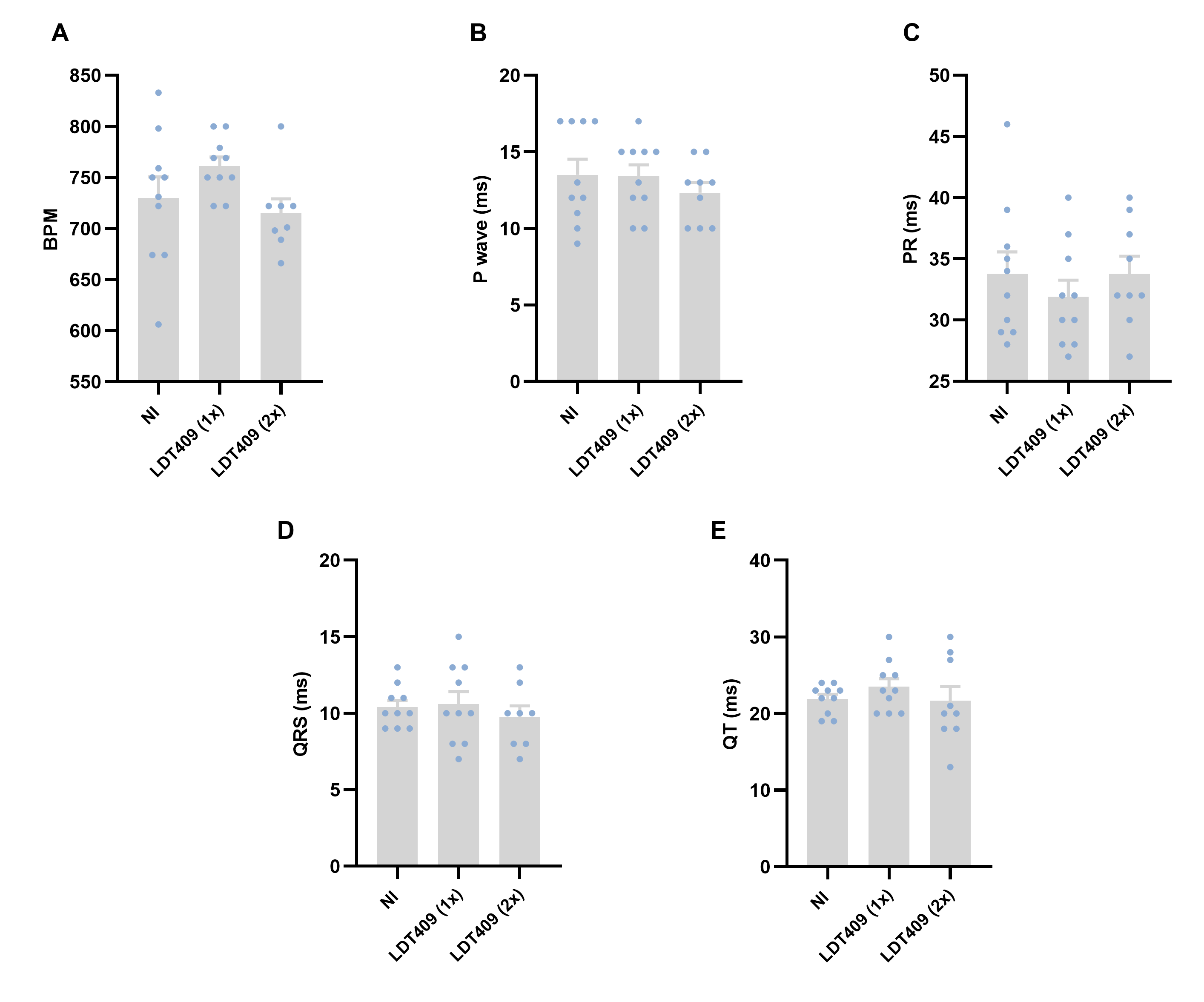

Supplement: Supplementary Figure 3 — Electrocardiographic analysis of non-infected animals treated with LDT409. (A) P wave (ms); intervals: (B) PR (ms); (C) QRS (ms); (D) QT (ms) and (E) heart rate, expressed as beats per minute (bpm). Parametric data were analyzed by one-way ANOVA followed by Fisher’s LSD post hoc test, while nonparametric data were analyzed using the Kruskal–Wallis test followed by Dunn’s post hoc test. [file Image3.tif]

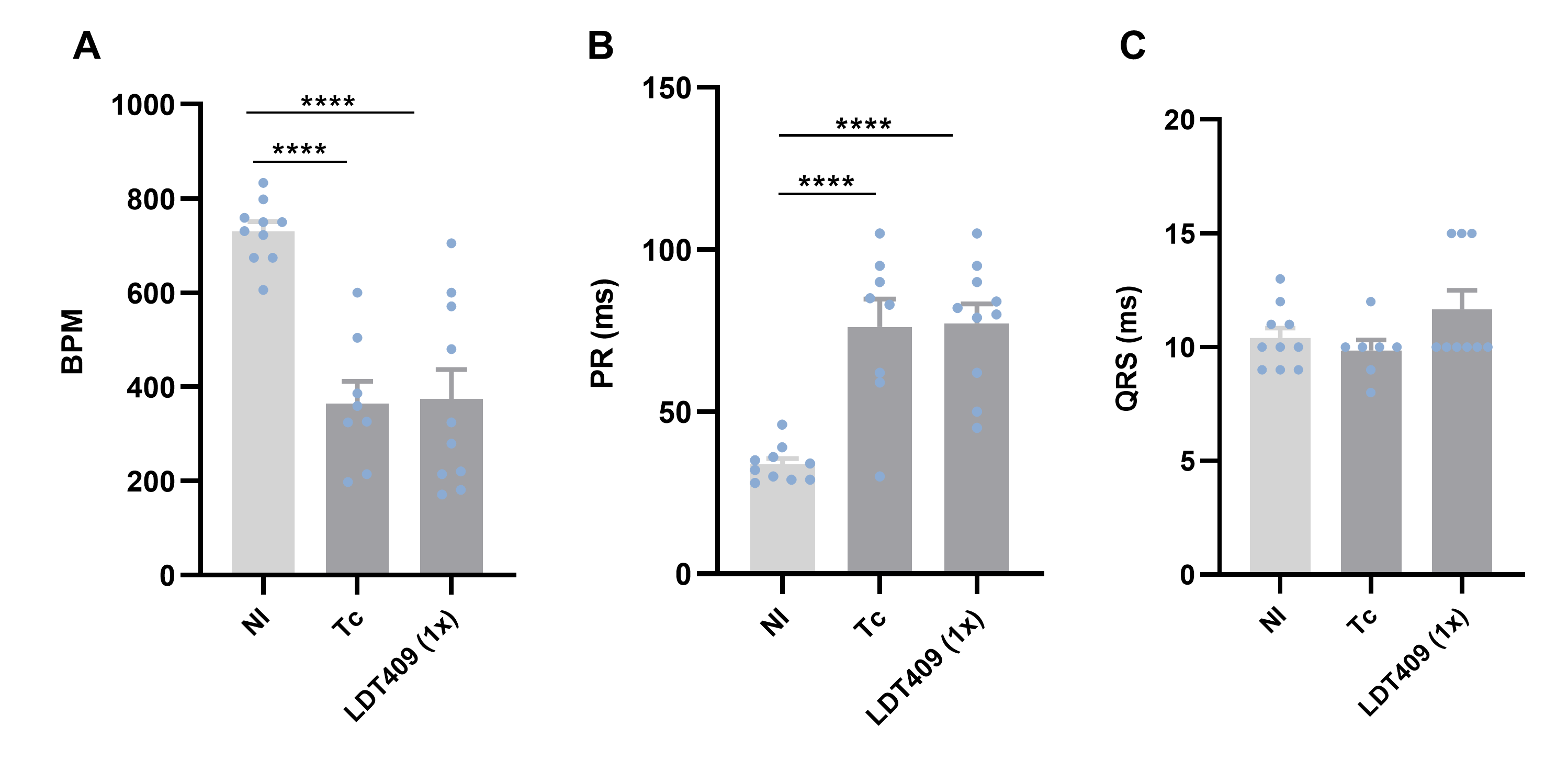

Supplement: Supplementary Figure 4 — Electrocardiographic analysis of T. cruzi-infected animals treated with LDT4091x. Intervals: (A) PR (ms); (B) QRS (ms); and (C) heart rate, expressed as beats per minute (bpm). Parametric data were analyzed by one-way ANOVA followed by Fisher’s LSD post hoc test, while nonparametric data were analyzed using the Kruskal–Wallis test followed by Dunn’s post hoc test. *, Different from NI. ****, P < 0.0001. [file Image4.tif]
