# Supplementary material for: Evaluation of the trypanocidal and immunomodulatory effects of LDT409, a cardanol derivative from cashew nut shell liquid
Source: Front Immunol. 2026 Mar 18;17:1749250. doi: 10.3389/fimmu.2026.1749250 (PMC13038549; doi:10.3389/fimmu.2026.1749250)
Supplement: Supplementary file 5 [file DataSheet1.pdf]

SwissTargetPrediction

Target	Common name	Uniprot ID	ChEMBL ID	Target Class	Probability*	Known actives (3D/2D)
G protein-coupled receptor 44 (by homology)	Ptgdr2	Q9Z2J6	CHEMBL2291	Family A G protein-coupled receptor	0.11573667475	129 / 28
Peroxisome proliferator-activated receptor delta (by homology)	Ppard	P35396	CHEMBL2458	Nuclear receptor	0.11573667475	100 / 40
Arachidonate 5-lipoxygenase (by homology)	Alox5	P48999	CHEMBL5211	Oxidoreductase	0.11573667475	82 / 32
Peroxisome proliferator-activated receptor alpha (by homology)	Ppara	P23204	CHEMBL2128	Nuclear receptor	0.11573667475	411 / 191
Epoxide hydratase (by homology)	Ephx2	P34914	CHEMBL4140	Protease	0.11573667475	30 / 0
Diacylglycerol O-acyltransferase 1	Dgat1	Q9Z2A7	CHEMBL1075284	Enzyme	0.11573667475	95 / 0
Group X secretory phospholipase A2 (by homology)	Pla2g10	Q9QXX3	CHEMBL4200	Enzyme	0.11573667475	5 / 0
Arachidonate 5-lipoxygenase-activating protein (by homology)	Alox5ap	P30355	CHEMBL3414408	Other cytosolic protein	0.11573667475	404 / 10
DNA topoisomerase I (by homology)	Top1	Q04750	CHEMBL2814	Isomerase	0.11573667475	2 / 0
Dual specificity phosphatase Cdc25B (by homology)	Cdc25b	P30306	CHEMBL2723	Phosphatase	0.11573667475	34 / 0
Matrix metalloproteinase-2 (by homology)	Mmp2	P33434	CHEMBL3095	Protease	0.11573667475	330 / 0
Serotonin 1a (5-HT1a) receptor (by homology)	Htr1a	Q64264	CHEMBL3737	Family A G protein-coupled receptor	0.11573667475	0 / 11
Prostanoid EP3 receptor	Ptger3	P30557	CHEMBL4336	Family A G protein-coupled receptor	0.11573667475	85 / 0
MAP kinase p38 alpha (by homology)	Mapk14	P47811	CHEMBL2336	Kinase	0.11573667475	5 / 2
Cholecystokinin B receptor (by homology)	Cckbr	P56481	CHEMBL2854	Family A G protein-coupled receptor	0.11573667475	175 / 0
MAP kinase ERK2 (by homology)	Mapk1	P63085	CHEMBL2207	Kinase	0.11573667475	4 / 1
Group IID secretory phospholipase A2 (by homology)	Pla2g2d	Q9WVF6	CHEMBL5537	Enzyme	0.0	2 / 0
Angiotensin-converting enzyme (by homology)	Ace	P09470	CHEMBL2994	Protease	0.0	98 / 0
Sn1-specific	Dagla	Q6WQJ1	CHEMBL5180	Enzyme	0.0	9 / 0

Target	Common name	Uniprot ID	ChEMBL ID	Target Class	Probability*	Known actives (3D/2D)
diacylglycerol lipase alpha (by homology)						
Multidrug resistance-associated protein 1 (by homology)	Abcc1	O35379	CHEMBL2532	Primary active transporter	0.0	3 / 0
Peroxisome proliferator-activated receptor gamma	Pparg	P37238	CHEMBL2459	Nuclear receptor	0.0	10 / 0
Glycerol-3-phosphate acyltransferase 1, mitochondrial	Gpam	Q61586	CHEMBL3580525	Transferase	0.0	3 / 0
Glucagon receptor (by homology)	Gcgr	Q61606	CHEMBL4773	Family B G protein-coupled receptor	0.0	66 / 0
Prostanoid DP receptor (by homology)	Ptgdr	P70263	CHEMBL3933	Family A G protein-coupled receptor	0.0	82 / 11
Cholecystokinin A receptor (by homology)	Cckar	O08786	CHEMBL2798	Family A G protein-coupled receptor	0.0	21 / 0
Transient receptor potential cation channel subfamily M member 8 (by homology)	Trpm8	Q8R4D5	CHEMBL3108632	Voltage-gated ion channel	0.0	10 / 0
Vasopressin V1a receptor (by homology)	Avpr1a	Q62463	CHEMBL3414410	Family A G protein-coupled receptor	0.0	9 / 0
Signal transducer and activator of transcription 3	Stat3	P42227	CHEMBL5402	Transcription factor	0.0	23 / 0
Neurotensin receptor 1 (by homology)	Ntsr1	O88319	CHEMBL3570	Family A G protein-coupled receptor	0.0	9 / 0
Nuclear receptor ROR-gamma (by homology)	Rorc	P51450	CHEMBL1293231	Nuclear receptor	0.0	2 / 0
Adenosine A1 receptor (by homology)	Adora1	Q60612	CHEMBL3688	Family A G protein-coupled receptor	0.0	1 / 0
Neurokinin 1 receptor (by homology)	Tacr1	P30548	CHEMBL2668	Family A G protein-coupled receptor	0.0	7 / 0
Anandamide amidohydrolase (by homology)	Faah	O08914	CHEMBL3455	Enzyme	0.0	3 / 0
Liver glycogen phosphorylase (by homology)	Pygl	Q9ET01	CHEMBL3008	Enzyme	0.0	40 / 0
Arachidonate 12-lipoxygenase (by homology)	Alox12	P39655	CHEMBL3225	Enzyme	0.0	0 / 7
Dopamine transporter (by homology)	Slc6a3	Q61327	CHEMBL2799	Electrochemical transporter	0.0	0 / 63
Estrogen receptor alpha (by homology)	Esr1	P19785	CHEMBL3065	Nuclear receptor	0.0	0 / 54
E3 ubiquitin-protein	Mdm2	P23804	CHEMBL3600279	Ligase	0.0	23 / 0

Target	Common name	Uniprot ID	ChEMBL ID	Target Class	Probability*	Known actives (3D/2D)
ligase Mdm2 (by homology)						
Caspase-1 (by homology)	Casp1	P29452	CHEMBL4800	Protease	0.0	108 / 0
Methionine aminopeptidase 2 (by homology)	Metap2	O08663	CHEMBL1075272	Protease	0.0	8 / 0
Prostanoid EP2 receptor	Ptger2	Q62053	CHEMBL2488	Family A G protein-coupled receptor	0.0	29 / 5
Cyclooxygenase-2 (by homology)	Ptgs2	Q05769	CHEMBL4321	Oxidoreductase	0.0	20 / 32
cAMP and cAMP-inhibited cGMP 3',5'-cyclic phosphodiesterase 10A (by homology)	Pde10a	Q8CA95	CHEMBL1795126	Phosphodiesterase	0.0	11 / 0
Prostanoid FP receptor	Ptgfr	P43117	CHEMBL5000	Family A G protein-coupled receptor	0.0	2 / 7
Thrombin (by homology)	F2	P19221	CHEMBL1075308	Protease	0.0	5 / 0
P-glycoprotein 3 (by homology)	Abcb1a	P21447	CHEMBL2573	Primary active transporter	0.0	0 / 73
P-glycoprotein 1 (by homology)	Abcb1b	P06795	CHEMBL3467	Primary active transporter	0.0	0 / 73
Cyclooxygenase-1 (by homology)	Ptgs1	P22437	CHEMBL2649	Enzyme	0.0	4 / 18
Monoamine oxidase A (by homology)	Maoa	Q64133	CHEMBL3681	Enzyme	0.0	0 / 17
Monoamine oxidase B (by homology)	Maob	Q8BW75	CHEMBL3050	Enzyme	0.0	0 / 32
Monoacylglycerol lipase ABHD6 (by homology)	Abhd6	Q8R2Y0	CHEMBL5010	Enzyme	0.0	1 / 0
Casein kinase II alpha (by homology)	Csnk2a1	Q60737	CHEMBL3537	Kinase	0.0	14 / 0
Casein kinase II alpha (prime) (by homology)	Csnk2a2	O54833	CHEMBL5326	Kinase	0.0	11 / 0
Macrophage colony-stimulating factor 1 receptor	Csf1r	P09581	CHEMBL5570	Kinase	0.0	5 / 0
Peripheral-type benzodiazepine receptor (by homology)	Tspo	P50637	CHEMBL2149	Membrane receptor	0.0	1 / 0
Eukaryotic translation initiation factor 4E (by homology)	Eif4e	P63073	CHEMBL6148	Other nuclear protein	0.0	9 / 0
11-beta-hydroxysteroid dehydrogenase 1	Hsd11b1	P50172	CHEMBL3910	Enzyme	0.0	9 / 9
Cannabinoid CB1 receptor (by homology)	Cnr1	P47746	CHEMBL3037	Family A G protein-coupled receptor	0.0	8 / 159

Target	Common name	Uniprot ID	ChEMBL ID	Target Class	Probability*	Known actives (3D/2D)
Cannabinoid CB2 receptor (by homology)	Cnr2	P47936	CHEMBL5373	Family A G protein-coupled receptor	0.0	7 / 169
Renin (by homology)	Ren1	P06281	CHEMBL2615	Protease	0.0	2 / 84
Thymidylate synthase (by homology)	Tyms	P07607	CHEMBL3160	Transferase	0.0	3 / 0
Serine/threonine-protein kinase 12 (by homology)	Aurkb	O70126	CHEMBL1075275	Kinase	0.0	9 / 0
Serine/threonine-protein kinase Aurora-A (by homology)	Aurka	P97477	CHEMBL2211	Kinase	0.0	7 / 0
Acyl-CoA desaturase 1 (by homology)	Scd1	P13516	CHEMBL5353	Enzyme	0.0	1 / 0
Ileal sodium/bile acid cotransporter (by homology)	Slc10a2	P70172	CHEMBL2073708	Electrochemical transporter	0.0	1 / 0
Ectonucleotide pyrophosphatase/ phosphodiesterase family member 2 (by homology)	Enpp2	Q9R1E6	CHEMBL3826871	Enzyme	0.0	5 / 0
Adenosine A2a receptor (by homology)	Adora2a	Q60613	CHEMBL2115	Family A G protein-coupled receptor	0.0	2 / 0
Norepinephrine transporter (by homology)	Slc6a2	O55192	CHEMBL2370	Electrochemical transporter	0.0	0 / 59
Dual specificity mitogen-activated protein kinase kinase 1 (by homology)	Map2k1	P31938	CHEMBL5860	Enzyme	0.0	0 / 1
Endothelin receptor ET-A (by homology)	Ednra	Q61614	CHEMBL2286	Family A G protein-coupled receptor	0.0	89 / 12
Matrix metalloproteinase 11	Mmp11	Q02853	CHEMBL3412	Protease	0.0	8 / 0
Tyrosine-protein kinase BTK (by homology)	Btk	P35991	CHEMBL3259478	Kinase	0.0	1 / 0
Protein-tyrosine phosphatase 1B (by homology)	Ptpn1	P35821	CHEMBL3336	Phosphatase	0.0	101 / 3
Plasminogen (by homology)	Plg	P20918	CHEMBL1075299	Protease	0.0	0 / 1
Urokinase-type plasminogen activator (by homology)	Plau	P06869	CHEMBL1075311	Protease	0.0	0 / 1
Dihydrofolate reductase	Dhfr	P00375	CHEMBL4564	Oxidoreductase	0.0	14 / 0
Protein-glutamine gamma-glutamyltransferase 2 (by homology)	Tgm2	P21981	CHEMBL2079853	Enzyme	0.0	1 / 0
Serine/threonine-	Mtor	Q9JLN9	CHEMBL1255165	Kinase	0.0	4 / 0

Target	Common name	Uniprot ID	ChEMBL ID	Target Class	Probability*	Known actives (3D/2D)
protein kinase mTOR (by homology)						
Serine/threonine-protein kinase B-raf (by homology)	Braf	P28028	CHEMBL2331061	Kinase	0.0	1 / 0
Serine/threonine-protein kinase TBK1 (by homology)	Tbk1	Q9WUN2	CHEMBL2189160	Kinase	0.0	2 / 0
Retinoid X receptor alpha (by homology)	Rxra	P28700	CHEMBL3084	Nuclear receptor	0.0	16 / 16
Serotonin transporter (by homology)	Slc6a4	Q60857	CHEMBL4642	Electrochemical transporter	0.0	0 / 87
Niemann-Pick C1-like protein 1 (by homology)	Npc1l1	Q6T3U4	CHEMBL1075296	Other membrane protein	0.0	1 / 0
Fatty acid synthase (by homology)	Fasn	P19096	CHEMBL1795189	Transferase	0.0	2 / 0
Calcitonin gene-related peptide type 1 receptor (by homology)	Calcrl	Q9R1W5	CHEMBL2034811	Family B G protein-coupled receptor	0.0	2 / 0
Sigma opioid receptor (by homology)	Sigmar1	O55242	CHEMBL3465	Membrane receptor	0.0	0 / 67
Vascular endothelial growth factor receptor 1 (by homology)	Flt1	P35969	CHEMBL3516	Kinase	0.0	1 / 0
Dihydroorotate dehydrogenase (by homology)	Dhodh	O35435	CHEMBL2991	Oxidoreductase	0.0	8 / 0
Glucose-dependent insulinotropic receptor (by homology)	Gpr119	Q7TQP3	CHEMBL5263	Family A G protein-coupled receptor	0.0	1 / 0
Hydroxycarboxylic acid receptor 2 (by homology)	Hcar2	Q9EP66	CHEMBL4420	Family A G protein-coupled receptor	0.0	4 / 0
Smoothened homolog (by homology)	Smo	P56726	CHEMBL6080	Frizzled family G protein-coupled receptor	0.0	1 / 0
Protein Wnt-3a (by homology)	Wnt3a	P27467	CHEMBL5617	Unclassified protein	0.0	1 / 0
Glycogen synthase kinase-3 beta (by homology)	Gsk3b	Q9WV60	CHEMBL1075321	Kinase	0.0	4 / 0
DNA-dependent protein kinase catalytic subunit (by homology)	Prkdc	P97313	CHEMBL2176779	Kinase	0.0	1 / 0
Tankyrase-2 (by homology)	Tnks2	Q3UES3	CHEMBL3232703	Enzyme	0.0	1 / 0
Insulin receptor (by homology)	Insr	P15208	CHEMBL3187	Kinase	0.0	1 / 0
C-C chemokine receptor type 2 (by homology)	Ccr2	P51683	CHEMBL5412	Family A G protein-coupled receptor	0.0	1 / 0

Target	Common name	Uniprot ID	ChEMBL ID	Target Class	Probability*	Known actives (3D/2D)
Retinoid X receptor beta	Rxrb	P28704	CHEMBL4047	Nuclear receptor	0.0	0 / 3
Glycogen synthase kinase-3 alpha (by homology)	Gsk3a	Q2NL51	CHEMBL2176843	Kinase	0.0	1 / 0
Omega-3 fatty acid receptor 1 (by homology)	Ffar4	Q7TMA4	CHEMBL2052036	Family A G protein-coupled receptor	0.0	1 / 0
